# Antiviral functions of Interferon-stimulated genes and virus-mediated antagonism

**DOI:** 10.3389/fimmu.2026.1961358

**Published:** 2026-10-23

**Authors:** Zhaobin Zhou, Yuchen Nan, Chunyan Wu

**Affiliations:** Department of Preventive Veterinary Medicine, College of Veterinary Medicine, Northwest A&F University, Yangling, Shaanxi, China

**Keywords:** interferons, interferon-stimulated genes, ISG15, JAK/STAT signaling, MX1, OAS, PKR, viral antagonism

## Abstract

Interferons (IFNs) are cytokines that play crucial roles in antiviral activity, anti-proliferation, stimulation of cytotoxic T cells, and modulation of host immune responses. Like other cytokines, IFNs bind their receptors on the cell surface to activate intracellular signal transduction. The major signaling pathway activated by IFNs is the Janus kinase/signal transducer and activator of transcription (JAK/STAT) pathway, which leads to the transcriptional activation of interferon-stimulated genes (ISGs). More than 300 ISGs can be upregulated by IFNs. Among these ISGs are a myriad of antiviral effectors, including ISG15, myxovirus resistance 1 (MX1), ribonuclease L (RNase L), and protein kinase R (PKR). Given the potent antiviral responses elicited by IFN-activated JAK/STAT signaling, viruses have evolved diverse strategies to antagonize this pathway during infection. In this review, recent progress in understanding the functions of ISGs is summarized, with particular emphasis on viral antagonism of ISG-mediated antiviral responses.

## 1 IFN-mediated signaling as an antiviral pathway

Interferons (IFNs) are a group of secreted cytokines that play key roles in host antiviral immunity, defined by their ability to inhibit virus replication. To date, three types of IFNs have been characterized (1–3). Type I IFNs, the largest IFN family, include IFN-α, IFN-β, IFN-ϵ, IFN-κ, and IFN-ω (4). All type I IFNs bind to a ubiquitously expressed heterodimeric receptor complex composed of IFN-α receptor 1 (IFNAR1) and IFNAR2 to activate the JAK/STAT pathway (5). Type II IFN consists solely of IFN-γ, which is classified separately from type I IFNs due to its distinct chromosomal localization and receptor usage (2, 6). Signaling for IFN-γ is transduced through the broadly expressed IFN-γ receptor complex (IFNGR) (7–9). IFN-γ plays a central role in establishing cellular immunity but is capable of inducing expression of a subset of antiviral genes that respond to type I IFNs as well (10, 11). Type III IFNs are the most recently identified family and comprise IFN-λ1 to IFN-λ4 (6, 12). The IFN-λs signal through a heterodimeric receptor composed of IFNLR1 (or IL-28R) and IL-10R2, which differs from type I/II IFN receptors. Moreover, the genes encoding IFN-λs are located at a distinct chromosomal locus (4, 6, 13). However, IFN-λs activate the same JAK/STAT pathway and induce gene expression patterns similar to those of type I IFNs.

Type I, type II and type III IFNs differ in the cells that produce them. Type I interferons (IFNs) are produced by most virus-infected cells, but plasmacytoid dendritic cells (pDCs) are the main producers of IFN-α *in vivo* (14). The type I interferon receptor is expressed on almost all cell types, which means type I IFNs can act throughout the body (1, 3). Type II IFN is produced mainly by activated T cells and natural killer (NK) cells rather than by infected cells, whereas its receptor is also widely expressed (2, 15). Type III IFNs are produced mainly by epithelial cells and their production is different between tissues. However, it is notable that the receptor of type III IFNs is expressed largely on epithelial cells (16). Therefore, type I and type II IFNs could act throughout the body whereas type III IFNs act only at mucosal surfaces and cause fewer systemic effects than type I IFNs (3, 17).

Like other cytokines, IFNs stimulate cells by binding to their corresponding receptors, thereby activating specific signaling pathways, principally the JAK/STAT pathway (18). Activation of JAK/STAT by IFNs triggers a cascade of events that ultimately leads to the induction of a broad set of IFN-stimulated genes (ISGs) (19) (**Figure 1**), commonly estimated at several hundred genes, whereas the exact number detected varies substantially among cell types and species (20, 21). These ISGs include antiviral effectors that restrict virus replication. In addition to the JAK/STAT pathway, activation of host pattern-recognition receptors (PRRs), including RIG-I-like receptors (RLRs) and Toll-like receptors (TLRs), also leads to ISG expression (22, 23). To date, considerable progress has been made in characterizing the cellular responses and antiviral activities mediated by ISGs. Genetic loss-of-function studies have identified major effector pathways or families of the interferon-mediated antiviral response, such as the MX GTPase pathway, the 2’,5’-oligoadenylate synthetase (OAS)-directed RNase L pathway, the protein kinase R (PKR) pathway, the ISG15 ubiquitin-like pathway, the tripartite motif (TRIM) family, and interferon-induced transmembrane proteins (IFITMs). These pathways collectively block viral transcription, degrade viral RNA, inhibit translation, and modify protein function to control viral replication. Ongoing studies continue to reveal additional activities of these effector proteins and uncover unexpected functions of antiviral responses (24). In this review, the functions of representative ISGs and viral strategies for antagonizing ISG-mediated antiviral responses are discussed in detail.

**Figure 1:**
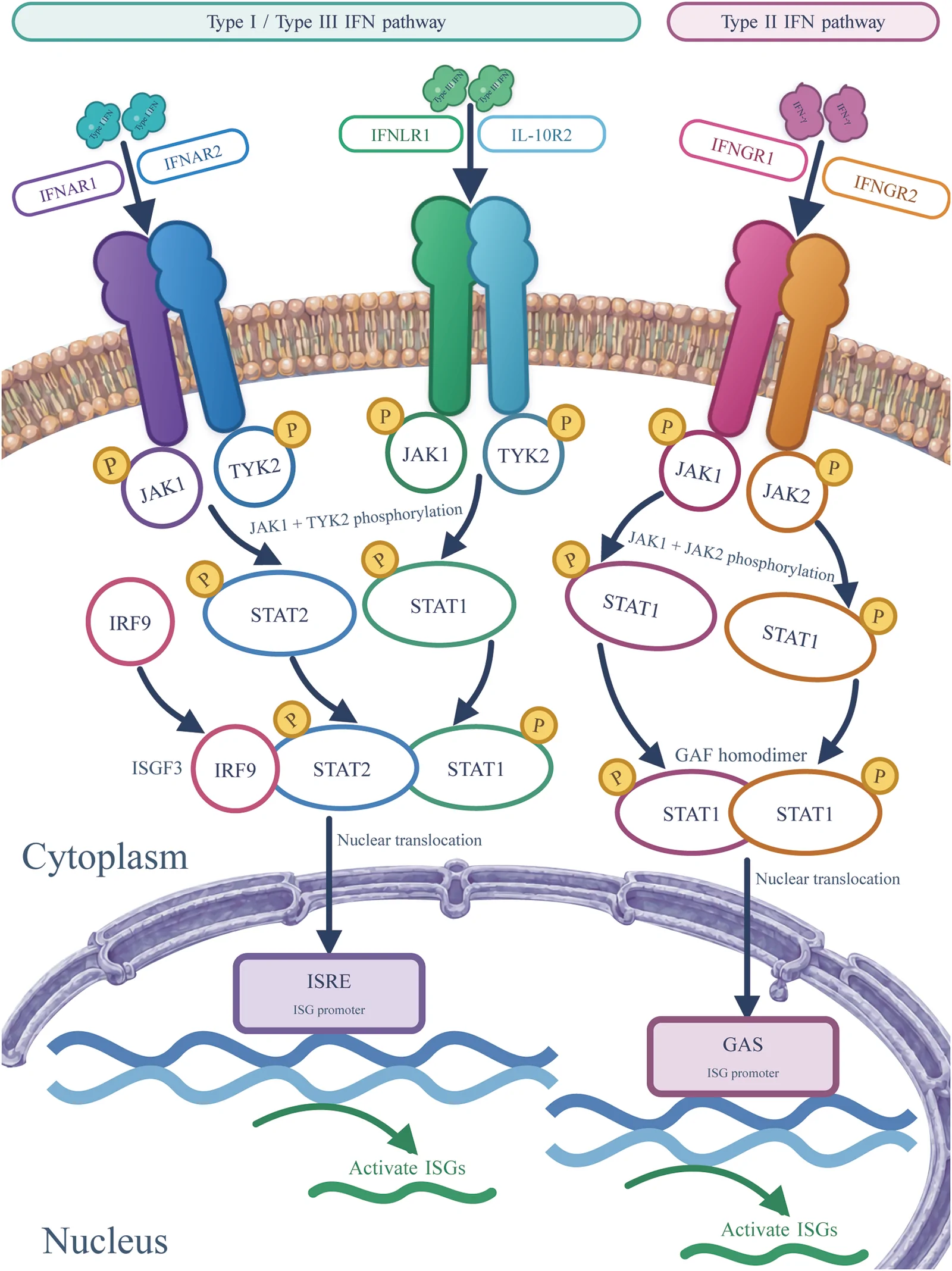
IFN signaling pathways that drive ISG expression. Binding of type I (IFN-α/β) and type III (IFN-λ) IFNs to their cognate receptor complexes (IFNAR1/IFNAR2 and IFNLR1/IL-10R2, respectively) activates JAK1/TYK2, leading to phosphorylation of STAT1 and STAT2, which associate with IRF9 to form the ISGF3 complex. Binding of type II IFN (IFN-γ) to IFNGR1/IFNGR2 activates JAK1/JAK2, leading to phosphorylation of STAT1 and formation of gamma-activated factor (GAF) homodimers. ISGF3 and GAF translocate to the nucleus, where they bind IFN-stimulated response elements (ISREs) and gamma-activated sequences (GAS), respectively, in the promoters of ISGs, thereby activating the transcription of hundreds of antiviral effector genes.

## 2 Functions of ISGs

The binding of type I IFNs to their receptors on the cell surface initiates a signaling cascade that leads to the induction of more than 300 ISGs. Some of these ISGs can also be upregulated by type II and type III IFNs (25, 26). The functional diversity of ISGs indicates that IFNs play multiple roles beyond their canonical antiviral or anti-tumor functions. Evidence from microarray studies has shown that ISG expression is cell type-specific, ranging from 50 to 1,000 genes, with 200 to 500 genes commonly expressed across cell types (25). This implies that IFNs can exert different functions in different cellular contexts. ISGs can be further classified into distinct categories according to their functions. Depending on the category, ISGs are involved in diverse pathways, including amino acid and lipid metabolism, antigen processing and presentation, host cell signaling, transcriptional regulation, ubiquitination, apoptosis, host defense, immune modulation, inflammation, and tumor suppression (26).

The ISGs that play important roles in antiviral defense during infection can be broadly divided into at least two groups based on their functions. The first group comprises proteins directly involved in host defense, including those that possess intrinsic antiviral activity at the cellular level. The second group includes ISGs that participate in host cell signaling. Within this group are PRRs, such as TLRs and RLRs, which detect pathogens and initiate host innate immune signaling. Certain adaptor proteins for TLRs that mediate signaling, such as MyD88, can be upregulated as well to enhance PRR signaling (27). Transcription factors, including members of the IRF family, the STAT family, and NF-κB, form amplification loops, thereby leading to enhanced IFN production and ISG expression (24, 26, 27). Moreover, proteins involved in other pathways can be upregulated, such as ubiquitin-activating E1 and ubiquitin-conjugating E2 enzymes, as well as IL-1β-converting enzyme (27). Among ISGs induced by IFNs, those acting as direct antiviral effectors are especially relevant to the outcome of infection, such as ISG15, MX1, the OAS family, RNase L, PKR and IFIT1 (**Figure 2**). Their prominence is supported by their participation in the virus-host arms race, as viruses have independently evolved antagonists against these ISGs, which indicates these effectors are important enough to shape viral evolution. In this section, each of these ISGs will be discussed.

**Figure 2:**
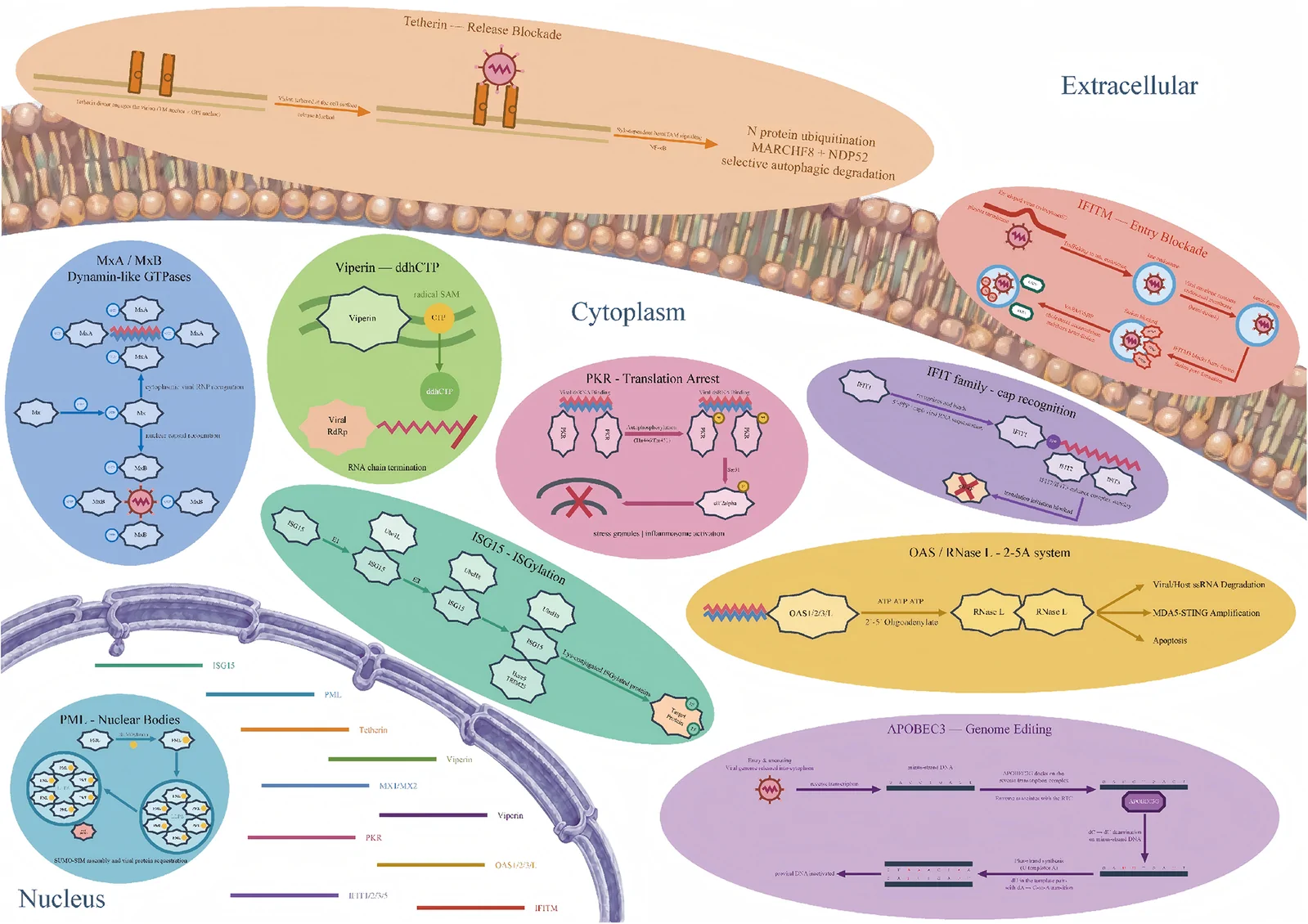
Antiviral effector mechanisms of the representative ISG families. Schematic illustration of the antiviral functions of the ten ISG families discussed in this review: ISG15 (ISGylation of viral and cellular proteins), MXA/MXB (dynamin-like GTPases that trap viral ribonucleoprotein complexes), the OAS/RNase L system (OAS1–3 synthesize 2’,5’-oligoadenylates (2-5A) that activate RNase L to degrade viral and host single-stranded RNA, with cleavage products amplifying MDA5/STING signaling and promoting apoptosis), the IFIT family (cap-dependent recognition and translational suppression of non-self RNA), PKR (double-stranded RNA-triggered translation arrest), viperin (a radical SAM enzyme that produces the chain-terminating nucleotide ddhCTP), PML nuclear bodies (nuclear scaffolds that sequester viral proteins and restrict virus replication), APOBEC3 proteins (cytidine deaminases that deaminate single-stranded viral DNA, leading to G-to-A hypermutation and degradation of the viral genome), IFITM proteins (intramembrane proteins that block fusion of enveloped viruses during cell entry), and tetherin (a GPI-anchored membrane protein that traps budding virions at the cell surface and prevents their release).

### 2.1 ISG15 and ISGylation system

ISG15 was identified following the discovery of ubiquitin and characterized as a potential ubiquitin homolog (28). The mature human ISG15 protein is a shorter product derived from a precursor by removal of eight amino acids at the C-terminus, thus sharing the identical C-terminal Leu-Arg-Leu-Arg-Gly-Gly sequence with mature ubiquitin (29). Unlike other ubiquitin homologs such as SUMO and Nedd8, ISG15 is not present in yeast, insects, plants, or other non-vertebrate organisms, implying that ISG15 performs a highly specialized function in vertebrates (30).

As a ubiquitin-like molecule, ISG15 can be conjugated to cellular substrates through a reversible process termed ISGylation, which involves E1-activating enzymes, E2-conjugating enzymes, and E3 ligases (28, 31). Protein ISGylation does not cause degradation of substrates, unlike K48-linked protein ubiquitination, and no universal role has been defined for protein ISGylation. It remains unclear whether ISGylated proteins face a common consequence analogous to ubiquitination, or whether their fates vary depending on the individual ISGylated protein (24, 32). However, evidence suggests that ISGylation triggers co-localization of multivesicular bodies (MVBs) with lysosomes and promotes the aggregation and degradation of MVB proteins in both HEK-293 cells and mouse primary macrophages (33).

The strength of ISG induction by IFNs does not always correlate with the potency of restriction or a particularly important role in viral antagonism. There are numerous examples of ISGs being extremely potent despite low to moderate levels of induction by IFN. However, the expression of ISG15 is strongly upregulated upon IFN treatment. Direct evidence for its role in host innate immunity comes from ISG15-deficient mice, which exhibit increased susceptibility to influenza A and B viruses, Sindbis virus and HSV-1 (34, 35). Conversely, mutation of the viral de-ISGylation machinery impairs viral replication; for instance, a point mutation impairing the protease activity of the ovarian tumor (OTU) domain in nsp2 of porcine reproductive and respiratory syndrome virus (PRRSV) results in reduced viral growth *in vitro* (36). ISG15 and ISGylation can target the budding of enveloped viruses (HIV-1, Ebola virus, Chikungunya virus) in a virus-specific manner (37). For example, ISG15 interacts with the ubiquitin E3 ligase Nedd4 and blocks Nedd4-mediated ubiquitination of Ebola virus VP40, resulting in reduced release of VP40 virus-like particles (38). However, a recent report also demonstrated that ISG15 deficiency restricts HIV-1 infection *in vitro* and it appeared that ISG15-deficient cells express elevated levels of other ISGs, consequently exhibiting broad-spectrum resistance to viral infection (39).

Conversely, the interference of ISGylation with ubiquitination blocks protein-protein interactions, suggesting a unique perspective for its function (32). ISG15 has been reported to interfere with the ubiquitination cascade (40), and can also prevent virus-mediated degradation of IRF3 to enhance IFN-β expression (41). Furthermore, ISGylation has been shown to modulate enzyme function. For example, conjugation of ISG15 to protein phosphatase 1B (PPM1B) suppresses its activity and enhances NF-κB signaling (42). In humans, free ISG15 binds to USP18 intracellularly, increasing its stability and leading to inhibition of IFN-α/β signaling; however, this phenomenon is not observed in mice (43).

In addition to its intracellular function as a protein modifier, a cytokine-like function of ISG15 has been proposed, as ISG15 can be secreted from human monocytes and lymphocytes and is detectable in both blood and urine (44, 45). It has been demonstrated that ISG15 stimulates the proliferation of NK cells as well as IFN-γ induction from T cells in a dose-dependent manner (44). Meanwhile, ISG15 also functions as a neutrophil chemotactic factor (45, 46). A recent study demonstrated that extracellular ISG15 activates PBMCs through the LFA-1 integrin receptor (CD11a) to enhance secretion of IFN-γ and IL-10 (47). Conversely, porcine ISG15 (pISG15) appears to exert antiviral activity in porcine alveolar macrophages (PAMs) against PRRSV infection when PAMs are pre-treated with recombinant pISG15 as a mimic for the extracellular form of ISG15 (48), although the underlying mechanism requires further investigation. In addition to its roles in viral infection, several reports have also demonstrated that aberrantly elevated or compromised ISG15 expression is observed in various human diseases, including multiple types of cancer, neurodegenerative disorders, inflammatory diseases, and bacterial infections (49, 50).

Recent studies have substantially expanded our understanding of ISG15 biology. A limited number of ISG15-specific E3 ligases have been described so far (51–53), which include HECT and RLD domain containing E3 ubiquitin protein ligase 5 (HERC5) in humans, HERC6 (mouse), ARIH1 and TRIM25 (53). Both HERC5 and HERC6 are themselves ISGs, forming a positive-feedback amplification loop (54, 55). The role of ISG15 during SARS-CoV-2 infection has also been actively investigated. ISGylation of the SARS-CoV-2 nucleocapsid (N) protein has been shown to impair its oligomerization and inhibit viral replication (56). Moreover, ISG15 has been demonstrated to directly activate MDA5 through ISGylation, enhancing the innate immune sensing of viral RNA independently of ubiquitin (57), representing a previously unappreciated mechanism of ISG15-mediated antiviral immunity.

### 2.2 The myxovirus resistance proteins

#### 2.2.1 MXA protein encoded by MX1

IFNs induce the expression of several guanine-hydrolyzing proteins, including the myxovirus resistance (MX) proteins. Humans possess two distinct MX genes located on chromosome 21, *MX1* (encoding MXA) and *MX2* (encoding MXB) (58). MX1 and MX2 were first identified as antiviral proteins through the observation that mutations within the *MX* locus on chromosome 16 confer susceptibility to orthomyxovirus infection in mice (59). Mouse strains expressing the active *MX1* gene (about 64% identity to human) are highly resistant to influenza virus infection (60). MX proteins have been classified as a subclass of the dynamin-like GTPase superfamily (61). Three domains have been identified from the N-terminus to the C-terminus of MX proteins: a GTPase domain, a central domain involved in protein-protein interaction, and the C-terminal effector domain with leucine zipper motifs (61).

The primary roles of MXA in humans or MX1 proteins in other mammalian hosts remain incompletely defined. MX1 in mice and rats is a nuclear protein (62), and has been demonstrated to inhibit influenza virus or influenza-like viruses such as Thogoto virus, as these viruses replicate in the nucleus. However, MXA or MX1 proteins from humans and other hosts are cytoplasmic proteins with smooth endoplasmic reticulum localization and have demonstrated broader inhibition of not only influenza virus but also other viruses such as vesicular stomatitis virus (VSV), Semliki Forest virus, and measles virus (62–65). Furthermore, although MX1 in mammalian hosts has been considered an inhibitory factor for influenza virus, the function of avian MX proteins differs. Only one MX protein with no antiviral activity has been identified in ducks and chickens (66, 67). However, later *in vitro* studies demonstrated that chicken MX protein is highly polymorphic, and the polymorphism at amino acid 631 is proposed to determine its antiviral activity (68, 69). Furthermore, chicken MX protein is dispensable for IFN-mediated resistance to influenza virus and lacks GTPase activity, which may explain its lack of antiviral activity (70). Although the detailed functions of MX proteins have not been fully defined, several virus-specific mechanisms have been identified. MX proteins can target viral nucleocapsid-like structures of Thogoto virus (71). The interaction between MX proteins has been characterized, and the smooth endoplasmic reticulum localization of MXs allows them to monitor exocytic events and mediate vesicle trafficking to trap essential viral components, thereby preventing viral replication at early time points (72). Moreover, MX1 can associate with subunits of the influenza virus polymerase to block viral transcription (73). Besides negative-strand RNA viruses, a recent study indicated that porcine MX1 plays a dominant role in inhibiting the N-Nsp9 interaction by interacting with the PRRSV N protein to inhibit replication of PRRSV (a positive-strand RNA virus) (74).

#### 2.2.2 MXB protein encoded by MX2

Unlike MX1, MX2 is localized in the nucleus due to a nuclear localization signal (NLS) within its N-terminus and is ineffective against influenza virus although MXB is highly homologous in sequence (63% identity) to MXA (75–77). Mouse lacks *MX2* gene, instead, mouse MXB protein is a duplicate of mouse *MX1* during evolution. Human MXB was shown to possess antiviral activity against HIV-1 by reducing the level of integrated viral DNA (78), with both nuclear accumulation and chromosomal integration of nascent viral complementary DNA suppressed (76, 79). It is notable that MXB mutants that are unable to bind or hydrolyze GTP retain the ability to restrict HIV-1 (76, 80). Consistent with these observations, MXB directly binds the HIV-1 capsid and this interaction depends on dimerization and the N-terminus of MXB (77), and transfer of MXB N-terminus residues 1–91 to MXA can confer potent anti-HIV-1 activity to human MXA (81). Moreover, a population-based analysis suggests that the MX2 haplotype is associated with resistance to HIV-1 infection in populations of Asian and European ancestry (82), further confirming the key role of MXB in inhibiting HIV-1 replication.

Besides HIV-1, MXB was recently shown to inhibit human herpesviruses in a GTPase-dependent manner by inhibiting the delivery of incoming HSV-1 DNA to the nucleus and the appearance of empty capsids (83, 84). A recent study demonstrates that MXB is packaged into the tegument of alphaherpesvirus virions and binds capsids via its N-terminal domain (85), but it remains unclear whether virion-associated MXB is capable of inhibiting herpesvirus replication or not. Moreover, MXB restricts the hepatitis viruses as well. Human MXB inhibits hepatitis C virus (HCV) replication in a cyclophilin A-dependent manner by binding the viral nonstructural protein NS5A and impairing its interaction with cyclophilin A and its localization to the endoplasmic reticulum (86, 87), and interferon-inducible MX2 restricts hepatitis B virus (HBV) transcription and replication in hepatoma cells and primary human hepatocytes (88). Similar to herpesviruses, GTPase activity is required for the restriction of HBV by MXB (88), whereas GTPase-deficient MXB mutants retain the ability to restrict HIV-1 (76, 80).

### 2.3 2’-5’ Oligoadenylate synthetase and RNase L

The 2’-5’ oligoadenylate synthetase (OAS) proteins and RNase L are two additional important ISGs that are functionally linked. Alternatively spliced terminal exons give rise to at least 8 isoforms, including five OAS1 isoforms (p42, p44, p46, p48 and p52), two OAS2 isoforms (p69 and p71) and one OAS3 isoform (p100), with OASL encoding 2 additional isoforms (89, 90). Depending on the catalytic domain, OAS1, OAS2, and OAS3 can form tetramers, dimers, and monomers, respectively (91). OAS proteins are unique in their capacity to synthesize 2’-to-5’-linked phosphodiester bonds to polymerize ATP into oligomers of adenosine (92). These unique 2’,5’-oligomers specifically activate the latent form of RNase L, which then mediates RNA degradation (93). Moreover, cyclic 2’-5’-linked RNA molecules can activate stimulator of interferon genes (STING) immune signaling as well (94). However, it appears that OAS3 is the major activator of RNase L, as OAS3 displays higher affinity for dsRNA in intact cells than either OAS1 or OAS2 in humans (95).

Human RNase L contains nine ankyrin repeats, several protein kinase-like motifs, and the RNase L domain from its N-terminus to the C-terminus (96, 97). The 2’,5’-oligomers bind to ankyrin repeats 2 and 4 and cause activation of RNase L (96, 97). RNase L monomers then form activated dimers to perform their RNase function (98). Although cellular RNA such as intact ribosomal RNA can be cleaved by RNase L (99, 100), data suggest that RNase L preferentially targets viral RNA and cleaves viral RNA at precise locations rather than random sites (101, 102). Moreover, viral RNA degraded by RNase L can then serve as a ligand for MDA5, resulting in enhanced induction of IFNs (93). The IFN induction mediated by OAS and RNase L is impaired in RNase L-deficient mice (93).

Recent studies have significantly advanced our understanding of the OAS/RNase L pathway, particularly in the context of human disease. Importantly, a Neanderthal-derived OAS1 haplotype has been associated with reduced COVID-19 severity, as this OAS1 variant encodes a prenylated isoform that localizes to endomembranes and more effectively senses SARS-CoV-2 RNA to activate RNase L (103, 104). A structural study using cryo-EM demonstrated that OAS2 Golgi localization is essential for dsRNA sensing and antiviral responses (105). Additionally, RNase L has been shown to promote autophagy as an antiviral mechanism, where RNase L-mediated RNA cleavage triggers autophagic degradation of viral components (106). The growing appreciation of the complexity of the OAS/RNase L pathway, including its roles in sterile inflammation and autoimmune disease, continues to expand the biological significance of this antiviral system.

In addition to OAS proteins, oligoadenylate synthetase-like protein (OASL) was identified as a new type of ISG closely related to the OAS family, characterized by an OAS-like domain at the N-terminus but devoid of 2’-5’ oligoadenylate synthetase activity in humans (107, 108). Unlike mice, which carry two functional OASL genes (mOASL1 and mOASL2), human OASL2 is a pseudogene (109). However, mOASL2 maintains OAS activity and requires dsRNA as an activator (110). Human OASL contains two tandem ubiquitin-like (UBL) domains in its C-terminus, which are unique within the OAS family (107, 108). Due to its inactive nucleotidyl transferase domain, OASL was initially assumed to interfere with the OAS-RNase L pathway by competing with OAS (110). Nevertheless, it has been demonstrated that human OASL possesses antiviral activity through its C-terminal UBL domain, particularly against RNA viruses (111). This is further supported by the observation that the antiviral activity of human OASL is independent of RNase L and is mediated by enhancing signaling of RIG-I (112). A recent study suggests that porcine OASL functions independently of the canonical RNase L pathway and interacts with MDA5 to mediate IFN induction and inhibit viral replication (113). However, the underlying mechanism for OASL-UBL domain-mediated RLR activation still requires further investigation.

### 2.4 Double-stranded RNA-dependent protein kinase

PKR is another important ISG that belongs to a small family of protein serine-threonine kinases that respond to environmental stresses to regulate protein synthesis (114). Two successive dsRNA-binding domains are located in the N-terminus of PKR, separated by a flexible linker region from the C-terminal kinase domain. PKR is constitutively expressed in all tissues at a basal level and can be upregulated by IFNs. Under normal circumstances, PKR is maintained as an inactive monomer. Activation of the cytosolic monomer is mediated by binding of viral double-stranded or structured single-stranded RNAs with a 5’-triphosphate end (115, 116). PKR activation causes dimerization and trans-phosphorylation of Thr446 (116). Activation of PKR following dimerization leads to phosphorylation of initiation factor eIF2α to halt mRNA translation. Therefore, PKR can promote signal transduction pathways, maintain homeostasis, mediate immune responses, and promote apoptosis after continuous activation through phosphorylation of protein substrates (117). However, it has been suggested that PKR could also be activated by cellular mRNA, but only when ribosomes are not initiating translation (118). Furthermore, PKR is involved in additional antiviral pathways, including promoting apoptosis, enhancing virus-induced IFN-β induction via regulating interferon mRNA integrity, and regulating inflammasome formation (119–121).

The integration of PKR into the broader stress response network has been increasingly appreciated in recent years. PKR has been shown to be a critical component of stress granule formation, where it interacts with stress granule-nucleating proteins such as GTPase-activating protein-binding protein (G3BP1) and T-cell intracellular antigen 1 (TIA-1) to promote the assembly of stress granules (122, 123). SARS-CoV-2 infection has been demonstrated to potently activate PKR, and the viral N protein antagonizes PKR-mediated stress granule formation, contributing to immune evasion (124, 125). Furthermore, it was reported that PKR physically interacts with multiple inflammasome components, including NLR family pyrin domain-containing 3 (NLRP3), NLR family pyrin domain-containing 1 (NLRP1), NLR family CARD domain-containing protein 4 (NLRC4), absent in melanoma 2 (AIM2), and broadly regulates inflammasome activation, which reveals a critical role of PKR in inflammasome activation (126).

In bacterial infections, PKR has emerged as a sensor as well. It can respond to bacterial RNA and secreted toxins, as well as promote selective autophagy to restrict the intracellular survival of pathogens such as *Mycobacterium tuberculosis* (127, 128). Moreover, novel PKR inhibitors developed through structure-based drug design are being explored as potential therapeutics for PKR-mediated inflammatory diseases such as neurodegenerative disorders associated with chronic PKR activation or inflammatory bowel disease (IBD) (129, 130). These recent discoveries underscore the multifaceted roles of PKR beyond canonical translation control and highlight its potential as a therapeutic target.

### 2.5 ISG56 and the IFIT protein family

ISG56 is a 56-kDa protein whose expression is extraordinarily responsive not only to IFN treatment but also to certain viral and bacterial molecular patterns. It is a member of the IFIT protein family (interferon-induced proteins with tetratricopeptide repeats), which comprises four members: ISG56/IFIT1, ISG54/IFIT2, ISG60/IFIT3/IFIT4, and ISG58/IFIT5 (131). The IFIT proteins are cytoplasmic and lack enzymatic activity but contain multiple copies of the eponymous tetratricopeptide repeat (TPR), a 34-amino-acid helix-turn-helix motif of degenerate sequence (132). Only nine residues in this motif show limited conservation, leading to differences in the primary structures of individual IFIT members (132). The TPR helix-turn-helix motifs mediate a variety of protein-protein interactions and exert diverse effects on cellular and viral functions, including translation initiation, virus replication, double-stranded RNA signaling, cell migration, and proliferation (131). The basal expression levels are low in most cell types, but IFIT proteins are highly IFN-inducible, and IFN treatment, viral infection, and activation of pathogen-associated molecular patterns (PAMPs) greatly enhance IFIT gene expression.

Although IFIT proteins lack known enzymatic activity, they interact with cellular and viral proteins through their multiple TPR motifs to form multi-protein complexes, thereby achieving a variety of cellular functions. The multifunctional tertiary structure of the TPR motif in IFIT proteins enables them to participate in diverse biological processes, including host innate immunity, antiviral immune responses, virus-induced translation initiation, replication, double-stranded RNA signaling, and PAMP recognition. Moreover, studies have shown that IFIT proteins are involved in tumor progression and metastasis as well (133). Experimental evidence from KSHV indicates that IFIT proteins can positively regulate the OAS-RNase L pathway to degrade RNA, and may directly interact with viral mRNA and cleave it to inhibit the replication of KSHV (134).

Current data support the notion that the IFIT family acts as translation inhibitors via binding to the 5’-terminal regions of cap0 (N7-methylguanosine), cap1 (m7GpppNmN), and 5’-ppp-mRNAs or 5’-PPP RNA (135, 136). However, *in vitro* data using purified IFIT proteins suggest selectivity of IFIT members for RNA with different caps (135). Crystallization of RNA-bound human ISG56 revealed that both N1 and N2 ribose 2’-O methylations of mRNA may play synergistic roles in self- versus non-self-mRNA differentiation (137). Conversely, the structure of ISG54/IFIT2 revealed that it binds specifically to certain RNAs, such as adenylate-uridylate (AU)-rich RNAs, with or without a 5’-PPP (136, 138). A recent study indicated that IFIT2 and IFIT3 form a domain-swapped heterodimer that is required for recognition of the viral mRNA 5’ end to inhibit translation (139), whereas viral or host 5’ UTR lengths less than 50 nucleotides are necessary and sufficient to enable translation inhibition by the IFIT2–IFIT3 complex (139). Human ISG58/IFIT5 differs from all other IFIT family members: it does not have a mouse homologue and is the only IFIT family protein identified in birds, marsupials, frogs, and fish so far (140). Structural analysis indicates that, similar to that of ISG54, the ISG58 structure can be divided into three regions and bears a “V”-like structure along with a spiral-like channel (141). However, ISG58 was found to copurify with a diverse mixture of small RNAs consisting mainly of a subset of tRNAs, and a number of noncoding RNAs (142).

Although the precise mode and specificity of RNA binding differ among IFIT proteins (143), implying a different substrate selectivity by IFIT members, IFIT1, IFIT2, and IFIT5 form superhelical structures containing a highly positively charged pocket for binding of different RNA ligands (135, 136, 144). Mutation of the positively charged residues within this pocket abolishes the RNA-binding property of IFIT members. Studies on Japanese encephalitis virus demonstrated that ISG56 preferentially binds to 5’-capped, 2’-O-unmethylated viral RNA, providing further evidence for the proposed function of IFIT1 (145). During the SARS-CoV-2 pandemic, it was discovered that the viral nsp16 2’-O-methyltransferase is essential for evading IFIT1-mediated restriction, and mutations that impair this activity render SARS-CoV-2 highly sensitive to IFIT-mediated translational suppression (146). Studies on parainfluenza virus type 5 (PIV5) have shown that ISG56 can limit the replication of viruses lacking 2’-O-methyltransferase activity, which modifies the 2’-hydroxyl group of ribose in the mRNA 5’-cap structure (147). Structural studies have further refined the mechanism by which IFIT1 discriminates between self and non-self RNA. The 5’ terminal m^6^A mark in the m^7^G cap had a protective function against IFIT1, which was additive with the effect observed for the 2’-*O* position (m^6^A_m_ cap-1). In contrast, an increased affinity for IFIT1 was observed for several noncanonical caps, including trimethylguanosine, unmethylated (G), and flavin-adenine dinucleotide caps, providing a precise structural basis for the long-standing observation of IFIT specificity (148).

Besides cap-related mechanisms, there is evidence suggesting that ISG56 inhibits translation by binding to specific subunits of eIF3, a mechanism of inhibition distinct from PKR and OAS (131). Translation of the hepatitis C virus (HCV) positive-sense RNA genome is initiated by internal ribosome entry site (IRES)-dependent ribosome recruitment, which differs from cap-dependent translation, and can be compromised in the presence of ISG56 (149, 150). For translation-unrelated inhibition, human ISG56 can directly bind the E1 helicase of human papillomavirus (HPV) to sequester E1 in the cytoplasm, separating it from the viral genome in the nucleus (151). Furthermore, ISG56 can also act as a negative-feedback regulator to dampen virus-induced innate immune signaling (152). Overexpression of ISG56 reverses the inhibition of VSV replication induced by cytoplasmic poly (I:C) (152), and competitive co-immunoprecipitation assays demonstrated that ISG56 disrupts the interaction between MITA and VISA or TBK1, two components of the virus-triggered IFN signaling pathway. These results suggest that ISG56 acts as a negative-feedback regulator of virus-triggered type I IFN induction and cellular antiviral responses (152). Moreover, besides recognition of RNA, IFIT3 was shown to repress adenovirus E1A immediate-early gene expression without blocking nuclear entry of the viral genome. Meanwhile, expression of IFIT3 restricts replication of human adenovirus by stimulating IFN-β production via the STING and MAVS pathways with the collaboration of its partner proteins IFIT1 and IFIT2, which reveals a novel antiviral mechanism of IFIT family members (153).

### 2.6 Viperin

Viperin is a highly conserved, 361-amino-acid protein with a molecular mass of 42 kDa, also known as RSAD2 (Radical S-adenosyl methionine domain-containing protein 2) due to significant homology with the MoA/PQQIII motif present in the radical S-adenosylmethionine (SAM) family of enzymes that use S-adenosylmethionine as a cofactor (154, 155). It was first identified as a human cytomegalovirus (HCMV)-inducible gene in fibroblasts and was renamed viperin (virus inhibitory protein, endoplasmic reticulum-associated, interferon-inducible) (156).

Radical SAM enzymes generally use the associated Fe-S cluster to generate a highly oxidizing deoxyadenosyl radical to mediate a variety of reactions (157). Although viperin binds Fe-S clusters like other radical SAM enzymes (157, 158), structures of viperin with a bound SAM analog or SAM cleavage products are consistent with a conventional mechanism of radical formation (155). Notably, sequence alignments guided by the putative active site residues of viperin reveal viperin-like enzymes in species from all kingdoms of life (155). Crystallization of viperin reveals that a portion of the active site has structural similarity to the GTP-binding site of molybdenum cofactor biosynthesis protein A and the RNA methyltransferase RlmN, which methylates specific nucleotides in rRNA and tRNA, suggesting viperin may recognize certain types of nucleoside triphosphates as substrates (155).

A landmark discovery in viperin biology has been the identification of its enzymatic product, whereby viperin catalyzes the conversion of cytidine triphosphate (CTP) to 3’-deoxy-3’,4’-didehydro-CTP (ddhCTP), a chain-terminating nucleotide that acts as a broad-spectrum antiviral by directly inhibiting viral RNA-dependent RNA polymerases (159, 160). Structural elucidation of the viperin active site with bound CTP and the Fe-S cofactor has provided atomic-level detail of the radical SAM mechanism underlying ddhCTP synthesis (155, 161), which is consistent with the previous observation that its Fe-S binding motif is essential for its functional activities in HCV and HCMV infection (162). Meanwhile, a recent study also suggested that viperin’s enzymatic product ddhCTP triggers ribosome collisions that stall translation (163). These findings represent a major breakthrough, as they assign a specific enzymatic function to viperin and explain its broad antiviral activity against both RNA and DNA viruses. Remarkably, prokaryotic homologs of viperin have been discovered in bacteria and archaea, which also produce antiviral nucleotide analogs, suggesting that this defense mechanism predates the evolution of the IFN system and represents an ancient, conserved antiviral strategy (164).

Current data suggest that, besides its enzymatic activity-related antiviral function, viperin employs a wide range of mechanisms, including inhibition of viral budding by affecting lipid rafts, and interaction with viral proteins in the case of influenza A virus and HCV, respectively (165). On the one hand, the antiviral effect of viperin requires endoplasmic reticulum (ER) association and appears to reduce the transport of soluble proteins from the ER, which might interfere with trafficking of critical viral components (166). On the other hand, viperin has been shown to inhibit influenza A virus and HIV-1 via blocking viral particle release (154). It also regulates cellular lipid metabolism during HCMV infection (167). This effect is achieved by affecting the biosynthesis of cholesterol. In a study of transient gene expression in human embryonic kidney 293T (HEK293T) cells, it was found that the expression of viperin could reduce the cholesterol level of these cells by 20% to 30% (168).

It is also suggested that viperin synergistically enhances the innate immune double-stranded DNA signaling pathway to limit virus infection. Viperin co-localized with the key signaling molecules of the innate immune dsDNA sensing pathway, STING and TBK1, binding directly to STING and inducing enhanced K63-linked polyubiquitination of TBK1 (169). Moreover, data obtained from pDCs demonstrate that viperin promotes TLR7- and TLR9-induced production of type I IFNs, further suggesting that viperin serves as an enhancer in IFN induction pathways as well (170).

### 2.7 Promyelocytic leukemia protein and TRIM family

The promyelocytic leukemia (PML) gene was first described as an oncogenic PML-retinoic acid receptor α (PML-RARα) fusion protein resulting from chromosomal translocation in acute promyelocytic leukemia (171). PML localizes to distinct nuclear multi-protein complexes termed ND10 or PML nuclear bodies (PML-NBs), which can be identified as electron-dense nuclear punctate areas within the interchromosomal regions of the nucleus (172). PML, also called TRIM19, belongs to the tripartite motif (TRIM) family, most of which are responsive to both type I and type II IFNs (173, 174). A later study demonstrated that the PML protein carries a conserved N-terminal arrangement of a RING finger, one or two B-box domains, and a coiled-coil region, and that the same three elements are present in a set of homologous proteins, which was designated the tripartite motif family, with *PML* as its founding member, TRIM19. Currently, more than 60 TRIM proteins are present in humans and mice, and a substantial subset is directly induced by type I interferons (174).

Since certain TRIM proteins have been demonstrated to exhibit ubiquitin E3 activity or SUMO E3 activity (175), a role for PML as an E3 ligase for SUMO modification has been proposed. Moreover, bioinformatics analysis based on a manually curated PML-NB interaction network retrieved from databases and literature reveals an association of all essential components of the SUMO conjugation pathway with PML-NBs, along with an enrichment of SUMOylated proteins at these sites (176). Furthermore, the proposed E3 ligase function for SUMO conjugation of PML is also consistent with a later report indicating that PML acts as an E3 ligase for SUMO modification of HCMV IE1p72 and mediates an intrinsic immune response against HCMV (175).

PML is expressed as seven isoforms, designated from PML-I to PML-VII, due to alternative splicing from a single gene, each with specific functions conferred by their C-terminal regions (177). The variability of the C-terminal part of PML isoforms is important for the recruitment of specific interacting partners and for the specific function. As demonstrated in PML knockout animals and cell lines, various reports have confirmed that PML is capable of inhibiting replication of both DNA and RNA viruses, such as HCMV, HSV-1, HIV-1, VSV, and influenza A virus, and even intracellular bacteria such as *Listeria monocytogenes* (178, 179). Studies of HIV-1 also indicated that although PML/TRIM19 does not restrict the early stages of HIV-1 infection (180), latent HIV-1 provirus resides in close proximity to PML nuclear bodies, whereas disruption of PML leads to reactivation of HIV-1 provirus in latent cells (181). However, the detailed mechanism of PML as an ISG was elusive for a long time and appears to require other PML-NB-associated proteins such as death domain-associated protein 6 (Daxx) (182).

Recent research has substantially advanced our understanding of PML-NB biology through the lens of liquid-liquid phase separation (LLPS). PML-NBs have been shown to exhibit properties of biomolecular condensates formed through LLPS (183), driven by the SUMO-SIM (SUMO-interacting motif) interaction network within the RBCC domain of PML (184, 185). This phase separation model provides a mechanistic framework for understanding how PML-NBs selectively sequester viral proteins and concentrate antiviral factors. This concept has been reinforced by the identification of viral ubiquitin ligases disrupting PML-NBs, similar to the mechanism employed by HSV-1 ICP0 (186, 187). Therefore, further investigation is needed to elucidate the exact role of PML as an ISG and to confirm whether the antiviral role of PML is SUMOylation-dependent.

As a nuclear protein, it remains unclear how PML inhibits RNA viruses whose replication occurs in the cytoplasm (188, 189). Among PML isoforms, only PML-VII lacks the nuclear localization signal (NLS) and exists as a cytosolic isoform. However, evidence from some RNA viruses suggested that PML-VII does not have antiviral activity against these RNA viruses (190). Currently, members of *Picornaviridae* are sensitive to PML-mediated antiviral activity. *PML* knockout mice are more sensitive to infection with encephalomyocarditis virus (EMCV), suggesting that PML is capable of inhibiting EMCV (191). Further research demonstrates that PML-IV, an isoform of PML, interacts with EMCV 3D polymerase (3Dpol) and sequesters it within PML-NBs to inhibit EMCV replication (192). Studies on enterovirus 71 (EV71), another member of Picornaviridae, indicated that PML repression of EV71 replication is partially mediated by the inhibition of autophagy, as PML deficiency triggered autophagy and promoted EV71 replication, similar to EMCV (193). Meanwhile, reports also demonstrated that VSV and rabies virus could be inhibited by PML-IV (190, 194), whereas PML-III also contributes to VSV inhibition (190). Besides directly preventing virus proliferation, it is suggested that PML can synergistically activate host IFN genes to further enhance innate immunity against viruses (179, 189), which also provides an explanation for its inhibition of RNA virus replication.

Besides PML (TRIM19), other TRIM members have also been identified as antiviral factors. *TRIM5α* was shown to restrict HIV-1 replication in Old World monkeys (194). *TRIM5α* restricts a broad range of retroviruses in a species-specific manner. It recognizes the HIV-1 capsid to induce premature uncoating and also engages the autophagy machinery through *ATG16L1* to activate innate immune signaling (194, 195). Besides SUMOylation, some TRIM proteins ubiquitinate both host and viral substrates as RING-type E3 ubiquitin ligases (174), such as *TRIM25*, which mediates K63-linked ubiquitination of the RIG-I CARDs and amplifies sensing of a wide range of RNA viruses (196). Meanwhile, *TRIM21* is a cytosolic Fc receptor that binds antibody-engaged virions and triggers antibody-dependent intracellular neutralization of nonenveloped and enveloped viruses (197), whereas *TRIM22* is an interferon-induced nuclear protein that suppresses HIV-1 particle production by blocking the budding of viral particles (198). Beyond antiviral signaling, TRIM proteins are also implicated in autophagy, development, and the regulation of protein homeostasis (174).

### 2.8 APOBEC3 family

The APOBEC (apolipoprotein B mRNA editing enzyme, catalytic polypeptide) superfamily started with APOBEC1, a cytidine deaminase that edits apolipoprotein B mRNA (199). However, since APOBEC3G was identified as an HIV-1 restriction factor that is antagonized by the viral Vif protein (200), and other members, including APOBEC3D, APOBEC3F, and APOBEC3H, were later shown to restrict HIV-1 and to be counteracted by Vif as well (201–204), it was suggested that members of the APOBEC superfamily could act as viral inhibition factors as well. Mechanistically, APOBEC3G interferes with reverse transcription by inducing numerous deoxycytidine-to-deoxyuridine mutations, which ultimately produces G-to-A hypermutation of the proviral DNA during HIV-1 replication (205, 206). Humans carry seven *APOBEC3* paralogs, from *APOBEC3A* to *APOBEC3*H (207). The crystal structure of the *APOBEC3G* catalytic domain shows a five-stranded β-sheet core with a zinc-coordinating active site, explaining the sequence-specific deamination of single-stranded DNA (208). All *APOBEC3* proteins contain one or two zinc-dependent cytidine deaminase domains. Members of *APOBEC3* are shown to inhibit replication of retroviruses, including human T-cell leukemia virus type 1 (209). In hepatocytes, interferon-induced APOBEC3 expression inhibits hepatitis B virus replication (210). *APOBEC3*-induced mutation of the HBV DNA genome occurs during reverse transcription of the viral RNA into minus-strand DNA (211), and *APOBEC3B* contributes to cccDNA decay under the control of NF-κB and *miR-138-5p* (212). It is notable that pigs carry a separate *APOBEC3* repertoire organized in multiple Z domains, which suggests independent expansion of the antiviral repertoire in the artiodactyl lineage (213). Porcine endogenous retroviruses show differential sensitivity to APOBEC3-mediated inhibition (214), and human APOBEC3 proteins repress PERV infection in heterologous systems (215).

Recently, the antiviral effects of *APOBEC3* proteins against herpesviruses have also been described. Human *APOBEC3* cytidine deaminases genetically edit herpes simplex virus 1 (HSV-1) and Epstein-Barr virus (EBV) genomes both *in vitro* and *in vivo*, with *APOBEC3C* overexpression reducing HSV-1 titers by about 10-fold and lowering the particle-to-PFU ratio (216), and HSV-1 R-loops serve as direct targets for APOBEC-mediated mutagenesis (217). Meanwhile, evidence suggests that human papillomaviruses (HPV) are edited by *APOBEC3* proteins as well. In a cohort study involving more than 5,000 HPV16-infected women, *APOBEC3*-induced mutations in the HPV genome were associated with viral clearance, whereas viral genomes from precancerous and cancerous lesions carried significantly lower mutation levels (218). Whole-genome sequencing of 156 HPV genomes covering 43 HPV types revealed highly variable APOBEC3 mutational signatures among HPV risk categories, suggesting that APOBEC3 editing levels are associated with the carcinogenic potential of HPV (219). Currently, there is no convincing evidence supporting restriction of RNA viruses by *APOBEC3*, although it has been reported that *APOBEC3G* and *APOBEC3H* interact with the nucleocapsids of human coronaviruses in an RNA-dependent manner (220, 221).

### 2.9 IFITM family

Interferon-induced transmembrane protein (IFITM) is another important ISG family. The human IFITM family comprises IFITM1, IFITM2, IFITM3, and IFITM5, whereas the numbering includes no IFITM4 because the original designations (9-27, 1-8D, 1-8U, and the bone-restricted BRIL) were reassigned (222). IFITM10 was first identified as a distinct vertebrate IFITM member in systematic phylogenetic analyses of the gene family (222, 223), in which the mammalian orthologs were defined. Functional studies of mammalian IFITM10 remain limited and largely outside antiviral immunity, with reported roles in tumor angiogenesis via STAT3 activation (224). IFITM proteins are highly hydrophobic membrane proteins of roughly 12 to 15 kDa mainly located in late endosomal and lysosomal membranes, and they bear two closely spaced intramembrane helices that are separated by a conserved intracellular loop (225). Generally, IFITM proteins are considered the first line of antiviral defense due to their unique function to block a broad range of membrane fusion events during infection by enveloped viruses (225), such as influenza A virus, West Nile virus, dengue virus, filoviruses, and coronaviruses (226, 227). IFITM3 has been extensively investigated for its antiviral mechanism during virus infection. IFITM3 restricts influenza A virus entry to permissive cells by blocking hemi-fusion and fusion pore formation between viral envelope and host membrane in late endosomes (228), a process that depends on IFITM3-mediated lipid sorting and stabilization of hemi-fusion (229). Meanwhile, IFITM3 interacts with the cellular cholesterol regulatory proteins vesicle-associated membrane protein A (VAPA) and oxysterol-binding protein (OSBP) to induce intracellular cholesterol accumulation, thereby blocking the membrane fusion process as well (230). Consistent with these functions, *Ifitm3*-deficient mice are susceptible to influenza A virus (231), whereas IFITM3 dysfunction led to early hypercytokinemia and predicted fatal infection in patients infected with avian-origin H7N9 influenza (232). IFITM proteins are also incorporated into HIV-1 virions, which reduces viral infectivity (233). In porcine species, IFITM proteins show broad antiviral activity as well (234). IFITM1 restricts porcine epidemic diarrhea virus (PEDV) infection *in vitro* (235), whereas swine IFITM3 delivered by recombinant Lactobacillus inhibits pseudorabies virus replication (236).

Conversely, it should be noted that *IFITM* proteins could be utilized by certain types of coronaviruses to enhance viral replication as well. Human coronavirus OC43 (HCoV-OC43) uses IFN-induced human IFITM2 or IFITM3 as an entry factor to facilitate its infection of host cells (237). Meanwhile, SARS-CoV-2 Spike protein interacts with IFITM1, IFITM2, and IFITM3 and hijacks them for efficient viral infection *in vitro* (238). A further study demonstrated that endogenous expression of IFITM2 and IFITM3 is required for efficient replication of SARS-CoV and SARS-CoV-2, whereas overexpression of IFITM2 and IFITM3 inhibits SARS-CoV and SARS-CoV-2 infection by relocalizing ACE2 (239). Mutations in the endocytosis-promoting YxxΦ motif of IFITM3 convert it into an enhancer of SARS-CoV-2 infection (240). Similarly, in porcine coronaviruses, IFITM proteins serve as key entry factors for PEDV (241). These findings suggest that IFITM proteins play a complicated role in virus replication and that their antiviral function might be virus-dependent or cell type-dependent.

### 2.10 Tetherin

Tetherin (also known as bone marrow stromal antigen-2, CD317, or HM1.24) was first identified as a surface antigen overexpressed on multiple myeloma cells (242). It is now recognized as an antiviral protein against many enveloped viruses. Tetherin is a type II single-pass transmembrane protein with a transmembrane anchor towards its N-terminus and a glycosylphosphatidylinositol (GPI) lipid anchor at its C-terminus, along with an extracellular coiled-coil domain (243). Tetherin forms parallel homodimers whose transmembrane domain and GPI anchor could physically tether nascent virions and retain virions at the cell surface (244, 245). A further study demonstrated that tetherin expression induces retention of virus-like particles (VLPs) assembled using the structural proteins of a range of enveloped viruses (246). Therefore, tetherin tethers a broad range of enveloped viruses, including retroviruses and filoviruses, and depleting or antagonizing it broadly increases virus release (243, 246). Beyond virion tethering, tetherin acts as an innate immune sensor. Retention of assembled virions at the plasma membrane induces NF-κB-dependent proinflammatory gene expression (247), and that signaling runs through a Syk-dependent hemITAM motif in the cytoplasmic tail (248). Besides retroviruses, herpes simplex virus 1 is inhibited by tetherin as well (249). Meanwhile, there was also a report demonstrating that porcine tetherin suppresses the replication of porcine enteric coronaviruses, including PEDV and swine acute diarrhea syndrome coronavirus (SADS-CoV) by binding and degrading the viral nucleocapsid protein (250). For PEDV, such degradation proceeds through selective autophagy coupled to N protein ubiquitination and can be blocked by autophagy inhibitors but not by a proteasome inhibitor, and it involves the E3 ubiquitin ligase MARCHF8 and the cargo receptor NDP52 (250). For SADS-CoV, tetherin reduces N protein expression through the same MARCHF8/NDP52 autophagosome pathway, and the tetherin transmembrane domain is essential for this activity (251). These findings suggest that tetherin might have additional ways to block virus replication.

## 3 Viral antagonism of ISG-mediated antiviral responses

Given the potent antiviral activities of ISGs, it is not surprising that viruses have evolved sophisticated countermeasures to evade or neutralize these effector pathways. Viral antagonism of ISG function can occur through multiple mechanisms, including direct cleavage or degradation of ISG proteins, inhibition of ISG enzymatic activity, sequestration of ISGs from their targets, and exploitation of ISG functions to benefit viral replication. Understanding these viral evasion strategies not only illuminates fundamental aspects of host-pathogen interactions but may also reveal vulnerabilities that can be exploited for antiviral drug development. In this section, selected examples of viral antagonism targeting each ISG effector pathway are discussed.

### 3.1 Antagonism of ISG15 and the ISGylation pathway

The antiviral functions of ISG15 and ISGylation have driven several viruses to evolve specific countermeasures. Since both ubiquitination and ISGylation rely on the terminal LRLRGG sequences of ubiquitin and ISG15 proteins and share a common conjugation mechanism, many viruses encode cysteine proteases with deubiquitinase activity that could act as de-ISGylating enzymes (252, 253), especially RNA viruses encoding proteases for the proteolytic processing of viral polyprotein precursors, such as *Arterivirus* (254), *Coronavirus* and *Picornavirus* (255, 256).

Based on homology and function, most viral cysteine proteases can be divided into two categories: papain-like proteases (PCPs) and ovarian tumor (OTU) proteases. Members of the order *Nidovirales*, including *Arterivirus* (e.g., PRRSV) and *Coronavirus* (e.g., SARS-CoV-2), generally encode both types of cysteine proteases (55, 257, 258). Beyond these viral families, the PCP domain of hepatitis E virus and the OTU domain of Crimean-Congo hemorrhagic fever virus (CCHFV) exhibit both deubiquitinating and de-ISGylating activities, thereby broadly suppressing innate immunity (259–261).

Besides viral proteases, other viruses employ different strategies to antagonize ISGylation. Influenza B virus employs its NS1 (NS1B) protein, which directly binds ISG15 and blocks the activation of the E1 enzyme UBE1L, thereby inhibiting the conjugation of ISG15 (262). Moreover, NS1B of influenza B virus could bind and sequester ISGylated viral proteins, primarily ISGylated viral nucleoprotein (NP), whereas ISGylated NP acts as a dominant-negative inhibitor of oligomerization of NP, thereby inhibiting viral RNA synthesis (263).

Conversely, intracellular conjugation of ISG15 negatively modulates its secretion. Viral de-ISGylases, including SARS-CoV-2 PL^pro^, positively modulate ISG15 secretion (264), which may promote ISG15’s signaling through the LFA-1 integrin to promote IFN-γ release from NK and T cells (264). The NS1B protein, previously reported to bind ISG15, blocked ISG15 secretion, suggesting that NS1B may function, in part, by inhibiting antiviral IFN-γ responses (264). Similarly, vaccinia virus E3 protein binds and antagonizes human and mouse ISG15 to inhibit ISGylation (265), whereas rotavirus non-structural protein 5 (NSP5) associates with UBE1L and targets UBE1L for proteasomal degradation to block ISGylation (266). Furthermore, human cytomegalovirus (HCMV) could downregulate ISG15 gene transcription through its IE1 protein to block ISGylation (267). Collectively, these examples demonstrate that viral proteases targeting the ISG15 conjugation pathway represent a broadly conserved immune evasion strategy.

### 3.2 Viral strategies to overcome MX protein restriction

Although MX proteins are broad-spectrum antiviral effectors against both DNA and RNA viruses (84, 268), certain viruses have evolved resistance mechanisms. Human myxovirus resistance protein A (hMXA) is a powerful gatekeeper that naturally targets the influenza NP protein. While avian influenza viruses are highly sensitive to MXA, human-adapted viral strains of avian influenza viruses carry specific, independent clusters of mutations in NP to evade this restriction. Influenza A viruses that have adapted to human hosts often carry mutations in the nucleoprotein (NP) that reduce susceptibility to MXA restriction (269–271). Specifically, amino acid residues in the NP of pandemic influenza A virus strains confer MXA resistance, contributing to the ability of these viruses to cross species barriers (269, 270, 272). The 1918 H1N1 pandemic influenza virus and highly pathogenic avian H5N1 and H7N7 influenza viruses possess MXA-resistant NPs, which likely contributed to their virulence in humans (269, 270, 272) and have been implicated in zoonotic transmission of avian and swine influenza A virus strains (273). Conversely, zoonotic IAV must acquire hMXA escape mutations to achieve sustained transmission in humans (273).

Unlike MXA (which targets cytoplasmic RNA viruses such as influenza), MXB acts at both viral entry and post-entry stages of the retroviral life cycle. Since MXB binds the HIV-1 core and inhibits the uncoating of the viral capsid (274), this interaction requires MXB oligomerization and depends on the N-terminal region of MXB as a capsid-binding motif (275, 276), and HIV-1 variants bearing capsid mutations such as G208R escape restriction by failing to bind MXB (277–279). Conversely, MXB restricts the nuclear import of the HIV-1 regulatory protein Rev and diminishes Rev-dependent expression of the HIV-1 Gag protein to inhibit HIV-1 replication. HIV-1 can overcome this inhibition by increasing the expression of multiply spliced viral RNA and hence Rev protein (280). However, the evolutionary arms race between MX proteins and viral nucleoproteins provides insight into the host specificity and pandemic potential of influenza viruses, as well as host susceptibility to HIV.

### 3.3 Viral countermeasures against the OAS/RNase L pathway

The OAS/RNase L system represents a formidable barrier to viral replication, and multiple viruses have evolved distinct strategies to circumvent this pathway. Influenza A virus employs its NS1 protein to inhibit OAS activation by competing with OAS for dsRNA binding through the dsRNA-binding characteristics of its RNA-binding domain (RBD), therefore effectively preventing the synthesis of 2’,5’-oligoadenylates and thereby blocking RNase L activation (281, 282). Vaccinia virus (VACV) encodes the E3L protein to prevent OAS activation (283), which may act through sequestering dsRNA, a key activator of OAS (284–286). Meanwhile, the VACV decapping enzymes D9 and D10 reduce dsRNA accumulation and thus also inhibit OAS/RNase L-mediated antiviral responses (287, 288). Theiler’s virus L* protein counteracts the OAS/RNase L pathway through direct interaction with the ankyrin domain of RNase L, resulting in the inhibition of this enzyme (289).

Rotaviruses and mouse hepatitis virus (MHV) have developed a different strategy: these viruses encode phosphodiesterases (PDE) that directly degrade 2’,5’-oligoadenylates (2-5A), the second messengers required for RNase L activation (290–292). The ns2 protein of MHV bears PDE activity to block the OAS-RNase L pathway (293). The C-terminal domain of rotavirus protein VP3 was identified as another potential PDE, which is evidenced by the ability of rotavirus VP3 to substitute for the function of ns2 in recombinant MHV (291, 294). Sequence alignments suggest that a similar PDE occurs in all group A and likely group B and C rotavirus strains as well (291, 294). Notably, a recent study suggested that viral PDEs encoded by nidoviruses and rotaviruses appear to be descended from host AKAP7-like PDE genes through horizontal gene transfer (295). In addition to viral PDEs, the SARS-CoV-2 nonstructural protein nsp15, an endoribonuclease (EndoU) universally present in coronaviruses, has been shown to evade the OAS/RNase L pathway by cleaving viral RNA to block activation of dsRNA-induced antiviral responses, which include the OAS and RNase L pathways (296, 297).

Besides viral protein-based strategies, certain viruses bear RNA elements capable of evading OAS/RNase L-mediated antiviral effects. As a typical example, RNase L cleaved HCV mRNA predominantly at UA and UU dinucleotides within loops of predicted stem-loop structures (298). HCV mRNAs from relatively interferon-resistant genotypes (HCV genotypes 1a and 1b) have fewer UA and UU dinucleotides than HCV mRNAs from more interferon-sensitive genotypes (HCV genotypes 2a, 2b, 3a, and 3b) (298). Meanwhile, poliovirus carries an RNA structure that acts as a competitive inhibitor (ciRNA) that inhibits the endonuclease activity of RNase L (299, 300). The human immunodeficiency virus (HIV) Tat protein binds to TAR, a dsRNA structure in the HIV mRNA, to prevent OAS activation by TAR (301).

Conversely, certain viruses could induce expression of the RNase L inhibitor to block the function of RNase L. HIV infection can inhibit the host’s antiviral RNase L pathway primarily by promoting expression of a cellular protein called ATP-binding cassette sub-family E member 1 (ABCE1) or RNase L inhibitor (302). Similarly, Encephalomyocarditis virus (EMCV) inhibits RNase L activity by promoting ABCE1 expression as well (303). However, a recent study suggested that ABCE1 may function as a positive regulator of exogenous RNA decay rather than an inhibitor of RNase L, while ABCE1 directly binds to form a complex with RNase L and accelerates RNase L dimer formation in the absence of 2’-5’ oligoadenylates (2-5A) (304). The diversity of these antagonistic strategies highlights the evolutionary pressure exerted by the OAS/RNase L pathway on viral replication.

### 3.4 Viral evasion of PKR-mediated translation inhibition

PKR represents a central node in the IFN-induced antiviral response, and accordingly, a remarkably diverse array of viral countermeasures has evolved to antagonize its function. Influenza A virus NS1 protein directly binds to and sequesters dsRNA, preventing PKR activation, and also interacts with PKR by a five-residue short peptide sequence to inhibit its kinase activity (282, 305–307). Vaccinia virus encodes two PKR antagonists, E3L and K3L. E3L sequesters dsRNA using its C-terminal dsRNA-binding domain (308). It is notable that the dsRNA-binding domain of E3L shares homology with the dsRNA-binding domain of PKR (308). E3L also directly binds to PKR to block its activation (309). K3L acts as a pseudosubstrate and competitive inhibitor of eIF2α phosphorylation (310). HSV-1 produces the ICP34.5 protein, which directly binds eIF2α to recruit protein phosphatase 1 (PP1) to dephosphorylate eIF2α, thereby reversing PKR-mediated translational arrest (311).

HCV NS5A protein interacts with and disrupts the dimerization of PKR through a direct protein-protein interaction involving the 40-amino-acid ISDR (interferon sensitivity-determining region) domain (aa 237–276) of NS5A (312, 313). Adenovirus counteracts PKR by expressing virus-associated RNA (VA-I RNA), a highly structured non-coding RNA that mimics dsRNA to bind to PKR but fails to activate it, thereby acting as a competitive inhibitor (314–316). Structural analysis suggested that while dsRNA allows multiple PKR monomers to line up side-by-side to cross-phosphorylate each other, the shape of the VA-I central domain pseudoknot distorts such architecture, which acts as a physical wedge that keeps the bound PKR molecule in an inactive, monomeric form (314, 316). The Epstein-Barr virus (EBV)-encoded small RNA (EBER1) shares strong functional, structural, and transcriptional similarities with adenovirus VA-I RNA as a competitive inhibitor of PKR (317, 318). However, a recent study has suggested that EBER1 binds PKR much more weakly than VA-I and exhibits weak PKR activation (319). The extraordinary diversity of viral PKR antagonists, encompassing RNA decoys, pseudosubstrates, dsRNA sequestration, phosphatase recruitment, and direct protein-protein inhibition, underscores the critical importance of PKR in antiviral defense.

### 3.5 Viral interference with IFIT-mediated translation restriction

The ability of IFIT proteins to discriminate between self and non-self mRNA based on 5’-cap methylation status has driven many viruses to evolve cap-modifying enzymes. In flaviviruses, the NS5 methyltransferase converts cap-0 into cap-1 by adding a 2’-O methylation to the first nucleotide, thereby protecting viral RNAs from IFIT1-mediated restriction (320–323). It is worth noting that a West Nile virus (WNV) mutant bearing NS5 E218A lacking methyltransferase activity is nonpathogenic in WT mice but is lethal in *Ifit1*^−/−^ mice (321). Similarly, coronaviruses, including MHV, SARS-CoV and SARS-CoV-2, encode 2’-O-methyltransferases within their nonstructural protein 16 (nsp16) that methylate viral RNA caps to evade IFIT1-mediated detection (324, 325). A recent study indicated that efficient 2’-*O* methylation in SARS-CoV-2 requires the assembly of the nsp16/nsp10 complex, in which the N-terminus of nsp10 is essential for efficient 2’-O methylation (326). However, the MHV mutant bearing the nsp16-D130A mutation, which lacks methyltransferase activity, is unable to replicate in the spleen of WT mice but replicates well in the spleen and macrophages of *Ifit1^−/−^* mice (324), whereas recombinant SARS-CoV-2 lacking 2’-O-methylation showed poorer replication efficiency but caused more severe disease in a mouse model (327), which may be a consequence of triggering a robust inflammatory response (325). Therefore, methylation of virus-derived RNA can affect more RNA sensors than IFIT1, which may be related not only to virus replication but also to inflammation-related pathogenesis.

Besides methylation of viral RNA, alphaviruses, such as Venezuelan equine encephalitis virus (VEEV), Sindbis virus (SINV) and Chikungunya virus (CHIKV), have evolved molecular strategies that involve the folding of a 5’ RNA structure that hides the cap from IFIT1-mediated recognition (328). Mechanistically, the first nucleotides of the alphavirus genomes are predicted to fold into a conserved 5’ stem-loop (5’SL) structure, thereby limiting the accessibility of the cap0 structure of the viral genome to IFIT1 (329). Moreover, another alphavirus member, Venezuelan equine encephalitis virus (VEEV), bears a hairpin secondary structure adjacent to the 5’ end of its RNA, which prevents IFIT1 from accessing the cap region of the viral RNA (330).

### 3.6 Viral proteins targeting viperin

Although viperin possesses broad antiviral activity, several viruses have developed strategies to antagonize its function. HCV employs its NS5A protein to interact with viperin to block synthesis of ddhCTP, thereby counteracting its antiviral effects (331). Meanwhile, coexpression of viperin with NS5A also reduces cellular NS5A levels, most likely by increasing the rate of proteasomal degradation of NS5A (331). Similarly, Japanese encephalitis virus (JEV), another member of the Flaviviridae like HCV, is capable of inducing proteasome-mediated degradation of viperin, whereas MG132 treatment could sustain the protein level and antiviral effect of viperin in JEV-infected cells (332).

Interestingly, some viruses appear to exploit viperin’s functions for their own benefit. Although viperin was originally identified during HCMV research as cytomegalovirus-induced human gene-5 (cig-5) (333), HCMV infection induces the expression of viperin and simultaneously relocalizes viperin from the ER to mitochondria, while viperin interacts with the mitochondrial trifunctional protein that mediates β-oxidation of fatty acids to reduce ATP generation, thereby benefiting viral replication (334). Similarly, since HIV-1 infection induces the expression of viperin, viperin supports sustained HIV-1 infection in macrophages, potentially through mechanisms involving the alteration of the LTR chromatin structure and the interferon response (335, 336). This dual role of viperin, antiviral in some contexts but proviral in others, exemplifies the complex and nuanced nature of virus-ISG interactions.

### 3.7 Viral antagonism of PML nuclear bodies and TRIM family

Both DNA and RNA viruses have evolved mechanisms to disrupt PML-NBs, underscoring the antiviral importance of these nuclear structures. For RNA viruses, an early report indicated that Z proteins from lymphocytic choriomeningitis virus (LCMV) and Lassa virus interact with the N-terminal region of PML and redistribute PML to the cytoplasm (337), which is supposed to block the function of PML, although no direct evidence has been provided. Meanwhile, as PML inhibits EV71 replication by inhibiting autophagy, it is countered by proteasome-independent degradation mediated by the viral protease 3Cpro (193). Similarly, EMCV 3Cpro leads to cleavage of PML to antagonize its antiviral effect (191). Meanwhile, a recent report indicates that the NS5 protein of Zika virus bears a SUMOylation site and that SUMO-conjugated NS5 disrupts the formation of antiviral PML-STAT2 NBs and directs PML degradation (338). Moreover, dengue virus NS5 complexes with PML isoforms III and IV to induce PML-NB breakdown as well (339).

HCMV expresses the immediate-early protein IE1, which directly binds to the PML coiled-coil domain and disrupts PML-NB formation through inducing a loss of PML SUMOylation (340). Meanwhile, although an early study indicated HCMV IE1 protein binds to PML and disrupts PML by preventing SUMOylation of PML (341), a recent study indicated that the HCMV mutant bearing an IE1 mutant lacking association with PML bodies replicates as efficiently as the wild-type virus, which challenges current views regarding the role of PML bodies in HCMV infection (342). Conversely, HSV-1 ICP0 protein contains a RING-type E3 ubiquitin ligase domain and a SUMO-interacting motif (SIM, aa362-364) and targets PML for proteasomal degradation (187, 343). ICP0 differentiates two similar substrates, PML isoforms I and II, and has profound regulatory effects on PML-II degradation (344).

Similar to HSV-1, varicella-zoster virus (VZV) ORF61 protein, an ICP0 homolog, also disrupts PML-NBs, whereas viruses bearing mutations of the ORF61 SIMs show impaired viral replication in skin (345). Adenovirus E4-ORF3 protein interacts with PML-NBs to antagonize the antiviral function of PML-NBs (346). Later research suggested that the E4-ORF3 protein could assemble into polymers and form a unique nuclear scaffold that leads to sequestration of SUMO (347, 348), providing further evidence for the previous observation that disruption of E4-ORF3 multimerization abolished its antagonism of PML-NBs (346). These diverse strategies for disrupting PML-NBs highlight the critical antiviral role of these nuclear bodies and the extent to which viruses have evolved to neutralize them. Moreover, porcine PML has been characterized and shown to restrict PRRSV replication, and the nsp1β protein of PRRSV, as well as its counterparts in the same viral family, antagonizes this restriction by inhibiting PML expression at the post-translational level (188).

Besides PML, viruses have evolved ways to counter other TRIM members as well. Influenza A virus NS1 binds *TRIM25*, blocks its association with *RIG-I*, and thus prevents the TRIM25-mediated ubiquitination of RIG-I that primes interferon induction (349). PRRSV nsp1α takes a degradative approach, targeting *TRIM25* for proteasomal degradation and suppressing host antiviral responses (350). PRRSV replication is restricted by *TRIM22*, which requires its nuclear localization signal for antiviral activity (351). In turn, PRRSV counters this restriction by upregulating host miR-376b-3p, which targets *TRIM22* (352).

### 3.8 Viral antagonism of APOBEC3 family

Since the antiviral functions of APOBEC3 family members were initially described for retroviruses, APOBEC3 antagonists were also first identified mainly in retroviruses. HIV-1 Vif is the prototypic *APOBEC3* antagonist (353). Vif recruits the CUL5-RBX2-ELOB-ELOC ubiquitin ligase complex through a *CBFβ*-dependent interaction, targeting *APOBEC3G* for proteasomal degradation and preventing its incorporation into virions (354). The structural basis of Vif-mediated antagonism has been defined at atomic resolution (355), and comparative studies reveal that HIV-1 Vif gained breadth in *APOBEC3G* specificity after cross-species transmission of its precursors (356), with the full structure-biology-evolution synthesis provided in a recent review (357). Although APOBEC3G has been extensively studied, HIV-1 Vif counteracts other APOBEC3 family members to achieve effective replication as well (204). A comprehensive analysis of the complete human APOBEC3 family demonstrated that APOBEC3D, APOBEC3F, and APOBEC3H are all potent restriction factors for HIV-1, whereas APOBEC3A/B/C have no significant impact on HIV-1 replication (204). APOBEC3F was identified as a second human antiretroviral factor that potently inhibits HIV-1 and is suppressed by the Vif proteins from both HIV-1 and HIV-2 (201, 202). Similarly, APOBEC3D restricts Vif-deficient HIV-1 and is counteracted by Vif as well (203, 204). However, the antiviral activity of APOBEC3H varies with the haplotypes since a deletion of Asn15 in haplotypes III and IV and an Arg105-to-Gly substitution in haplotype I abolished or attenuated APOBEC3H activity against HIV-1 in the majority of humans (358), whereas the stable variant, APOBEC3H haplotype II, retains potent activity against HIV-1 and remains common in African populations (358, 359). Accordingly, Vif proteins from different HIV-1 subtypes show distinct activity spectra against APOBEC3G, APOBEC3F, and APOBEC3H, and HIV-1 Vif adaptation to APOBEC3H haplotypes has been documented (359, 360). These observations indicate that Vif must antagonize a combination of APOBEC3D, APOBEC3F, APOBEC3G, and APOBEC3H, rather than APOBEC3G alone, to support optimal viral replication.

Besides HIV-1, retroviruses from other mammalian hosts also encode Vif proteins that act as APOBEC3 antagonists (361, 362). Vif in primate and feline lentiviruses degrades host APOBEC3 proteins in a species-specific manner (362), and feline immunodeficiency virus (FIV) protease is capable of antagonizing feline *APOBEC3* as well (361). Besides the Vif protein, foamy virus Bet proteins function as novel inhibitors of the *APOBEC3* family of innate antiretroviral defense factors. Foamy virus Bet can directly bind *APOBEC3* and impair its dimerization and cytosolic solubility (363, 364). Since mouse retroviruses do not encode a Vif homologue, the murine leukemia virus glycosylated Gag protein inhibits the antiretroviral function of *APOBEC3* as well (365), and a structural and functional study shows that glyco-Gag blocks apolipoprotein B editing complex 3 and cytosolic sensor access to the reverse transcription complex (366).

As recent studies have demonstrated that herpesviruses can be targeted by *APOBEC3* proteins, more *APOBEC3* antagonists have been identified in different herpesvirus families. Epstein-Barr virus *BORF2*, the large subunit of its ribonucleotide reductase, inhibits cellular *APOBEC3B* to preserve viral genome integrity (367). Meanwhile, ribonucleotide reductase large subunits of HSV-1, HSV-2, EBV and KSHV possess a conserved mechanism to relocalize *APOBEC3B* from the nucleus to the cytoplasm, where it can no longer access viral DNA (368–370). On the other hand, KSHV ORF61, a related R1 protein, sequesters *APOBEC3B* in filamentous cytoplasmic aggregates (371). Human cytomegalovirus mediates *APOBEC3B* relocalization early during infection through a ribonucleotide reductase-independent mechanism (372). Moreover, human herpesvirus 6B (*HHV-6B*) expresses high levels of the U28 protein, which binds multiple *APOBEC3* proteins and promotes their relocalization and degradation, whereas *HHV-6A* fails to counteract *APOBEC3* and consequently accumulates extensive mutations in both cell- and patient-derived genomes, providing a molecular basis for the divergent pathogenicity and evolvability of the two *HHV-6* species (373).

### 3.9 Viral antagonism of IFITM family

The best-characterized viral protein directly antagonizing an IFITM family member is HIV-1 Nef (374). Nef is a multifunctional accessory protein that downmodulates CD4 and MHC class I from the cell surface and boosts virion infectivity by counteracting SERINC3 and SERINC5. Its activity against IFITM3 is independent of these pathways. Nef was found to interact with IFITM3 in membranes, reduce IFITM3 incorporation into virions, and restore fusion of HIV-1 with target cells (374). Meanwhile, expression of Nef changes the proteome of T cell-derived extracellular vesicles and depletes IFITM1, IFITM2, and IFITM3 from them by excluding these proteins from the plasma membrane and lipid rafts, which limits the cell-to-cell transfer of this antiviral cargo (375). Moreover, viral resistance to *IFITM* is not limited to direct protein antagonism. HIV-1 variants transmitted to new hosts show resistance to *IFITM*-mediated restriction (376), and mammalian *IFITM* proteins differ in their activity against HIV-1, indicating that virus strains and family members interact in a strain-specific manner (377).

Besides directly antagonizing IFITM proteins, post-translational mechanisms that regulate IFITM expression appear to be involved in virus replication as well. ABHD16A functions as a depalmitoylase that removes S-palmitoyl groups from IFITM proteins, a modification required for IFITM clustering and antiviral activity (378). Therefore, ABHD16A-mediated depalmitoylation attenuates the antiviral function of IFITM proteins (378). Consistent with this, a recent report indicated that ABHD16A negatively regulates the anti-hepatitis B virus activity of IFITM1 during HBV replication (379). Together, these findings show that the antiviral potency of IFITM proteins is controlled not only by expression levels but also by post-translational modification and subcellular localization, which are natural points of intervention for viruses seeking to overcome IFITM restriction.

### 3.10 Viral antagonism of tetherin

During the discovery of tetherin as an HIV-1 restriction factor, the HIV-1 Vpu protein was identified as the first tetherin antagonist. Most viral antagonists of tetherin were identified from retroviruses. It is also notable that HIV-1 Vpu counteracts tetherin in a species-specific manner. Vpu overcomes human tetherin but not the rhesus macaque ortholog (380), and the specificity maps to amino acid differences in the transmembrane domain of tetherin. In contrast, SIV Nef downregulates cell-surface expression of rhesus tetherin but does not downregulate human tetherin, whereas the specificity of SIV Nef for rhesus tetherin is determined by four amino acids in the cytoplasmic domain of the molecule that are absent in human tetherin (380). Vpu antagonism depends on the adaptor protein AP-1 and on human-specific adaptations that are critical for efficient early HIV-1 replication (381, 382), and HIV-1 group M Nef proteins also possess tetherin antagonism (383), as does an HIV-1 group O Vpu variant (384). HIV-2 envelope glycoprotein shows incomplete *BST-2* antagonism and modulates NF-κB signaling (385), and a neurovirulent SIV envelope exhibits enhanced antagonism (386). Feline immunodeficiency virus envelope glycoproteins antagonize feline tetherin through a distinctive mechanism that requires virion incorporation (387), and the FIV envelope signal peptide is itself a tetherin-antagonizing protein (388). Equine tetherin blocks virion release of equine infectious anemia virus (EIAV), but its activity can only be antagonized by the EIAV envelope protein rather than by HIV-1 Vpu (389).

Besides retroviruses, the Ebola virus glycoprotein directly antagonizes tetherin-mediated restriction of filovirus budding. The Ebola GP interacts with tetherin (390), and GP can even substitute for HIV-1 Vpu to promote HIV-1 virion release from tetherin-expressing cells, indicating a common cellular target for these divergent viral proteins (390). Moreover, the SARS-CoV accessory protein ORF7a inhibits tetherin-mediated virion tethering by blocking the glycosylation of BST-2, and loss of ORF7a leads to much stronger tetherin restriction of SARS-CoV (391). The SARS-CoV-2 ORF7a also neutralizes BST-2, which inhibits viral egress at intracellular membranes (392), and Omicron spike mutations allow more efficient counteraction of tetherin restriction (393). Furthermore, Kaposi sarcoma-associated herpesvirus K5, a viral member of the membrane-associated RING-CH ubiquitin ligase family, ubiquitinates lysine residues in the short N-terminal domain of tetherin after the protein exits the endoplasmic reticulum, leading to rapid degradation and removal of tetherin from the cell surface (394). Herpes simplex virus 1 is restricted by tetherin and is antagonized by glycoprotein M (gM), which efficiently removes tetherin from the plasma membrane and can functionally substitute for the HIV-1 Vpu protein (249). These data suggest that herpesviruses use both ubiquitination and glycoprotein-based strategies.

## 4 Co-evolution of ISG function and viral antagonism

The ISG families discussed above are not a fixed defense system. Comparative analysis of proteins that interact with viruses, a much larger category than the classical ISGs, shows that viruses are a major cause of protein adaptation in mammals, and account for nearly 30% of all adaptive amino acid changes in the conserved part of the human proteome (395). Since an ISG inhibits the viruses it targets, a viral variant that is not inhibited replicates better, which in turn results in a host variant that restores the restriction. This type of reciprocal selection increases the diversity of both the host ISG and the viral antagonist, and both show a signal of repeated positive selection (396, 397).

Consistent with this model, positive selection has been identified in several ISG families, including IFIT1 (398, 399), the OAS family (400), and the TRIM family (401). Moreover, viperin has evolved under positive selection in primates, but functional analysis showed that lentiviruses are not the source of that selection, as viperin does not inhibit most strains of HIV-1 or other retroviruses (336). The selection that acts on an ISG therefore depends on which viruses the host is exposed to, rather than the range of viruses that the ISG can inhibit.

The most studied cases are those of the primate lentiviruses. Primate tetherin shows clear signs of positive selection, and HIV-1 Vpu has adapted specifically to antagonize the tetherin variants of humans and chimpanzees, and swapping the transmembrane domains between human and monkey tetherin changes their sensitivity to Vpu (402). Tetherin antagonism by Vpu and Nef is also different between pandemic and other HIV-1 groups, and the accessory protein used depends on the tetherin variant of the host (403). For the APOBEC3 family, Vif must antagonize the APOBEC3 repertoire of the host, including APOBEC3D, APOBEC3F, APOBEC3G, and APOBEC3H (204, 360), and the adaptation required for this antagonism in humans is considered an essential factor for the cross-species transmission of lentiviruses from monkeys to chimpanzees and then to humans (404). Similarly, the kinase domain of PKR shows the same pattern and has evolved rapidly, since the residues of PKR that bind viral antagonists are under positive selection. Therefore, inhibitors from different poxviruses differ in their ability to block PKR from different mammals (405, 406).

Conversely, species-specific ISG repertoires also contribute to species barriers to viruses with the ability to cause cross-species infection, since different hosts are exposed to different viruses, and each species has a different set of ISGs. Independent losses of OAS genes have been described in different mammals (407), the number of IFITM genes is also different among vertebrates (222), and the cytoplasmic RNA sensor MDA5 has been lost in certain avian species and is also not present in pangolins (408, 409). These differences contribute directly to the limited host range of many viruses, because a viral antagonist is generally specific for the ISG variant of its natural host and often cannot antagonize the corresponding ISG in a new host species. As a typical example, the host range of influenza A virus is determined by interactions between viral and host cell factors that differ between species, including the ability to evade host restriction factors (410), and a bat H9N2 virus is resistant to human MXA and shows efficient replication in ferrets (411). Meanwhile, for primate lentiviruses, SIVsmm from sooty mangabeys has crossed into humans on several occasions to give rise to HIV-2, and human APOBEC3 proteins restrict it more strongly than they restrict the HIV-2 strains that spread in humans (412). Therefore, the set of ISGs in a species is determined by the viruses it is exposed to. Each virus and its host interact in a different way, and most viruses are unable to infect new hosts because of these differences.

## 5 Conclusions and future perspectives

IFNs induce hundreds of ISGs through JAK/STAT signaling to establish an antiviral state. These ISGs include several ISG families, such as ISG15, MX, OAS/RNase L, PKR, IFIT, viperin, TRIMs, APOBEC3, IFITM and tetherin. The major function of these ISGs is generally considered to directly inhibit viral replication at various stages. Accordingly, viruses have evolved specific countermeasures to antagonize these ISGs as a survival strategy (**Table 1**). However, advances in recent years reveal new concepts that reshape our understanding of the IFN system and function of ISGs. Beyond their classical roles as direct antiviral effectors, ISGs are actively participating in the regulation of both IFN induction and activation of downstream JAK/STAT signaling. For example, viperin directly binds STING and TBK1 to enhance K63-linked polyubiquitination of TBK1, thereby amplifying the cGAS-STING-mediated IFN induction pathway (169). Similarly, OASL enhances RIG-I signaling through its C-terminal UBL domain independently of the canonical RNase L pathway (112, 113), and IFIT3 stimulates IFN-β production via the STING and MAVS pathways in collaboration with IFIT1 and IFIT2 (153). PKR, in addition to its translation-inhibitory function, broadly regulates inflammasome activation through physical interaction with NLRP3, NLRP1, NLRC4, and AIM2 (126). Conversely, certain ISGs function as negative-feedback regulators of IFN responses: ISG56 disrupts the MITA-VISA/TBK1 interaction to dampen virus-triggered type I IFN induction (152), while free ISG15 stabilizes USP18 to inhibit IFN-α/β signaling (43). These findings reveal a complex regulatory network in which ISGs themselves modulate the amplitude and duration of IFN responses, adding a layer of control beyond the classical receptor-to-nucleus signaling paradigm.

**Table 1:** ISG effector pathways and viral countermeasures.

ISG (effector function)	Virus	Viral factor	Antagonism mechanism	Refs.
**ISG15/ISGylation** *ISGylation of viral and cellular proteins; blocks enveloped-virus budding (e.g., Ebola VP40); impairs SARS-CoV-2 N oligomerization; activates MDA5; secreted ISG15 signals via LFA-1*	PRRSV	nsp2 OTU protease	Cleaves ISG15 from substrates (de-ISGylation)	(36, 252–254)
	SARS-CoV-2	PLpro	de-ISGylation; enhances ISG15 secretion	(264)
	CCHFV	OTU protease	deubiquitinating and de-ISGylating activities	(259–261)
	HEV	PCP protease	deubiquitinating and de-ISGylating activities	(259–261)
	Influenza B virus	NS1B	Binds ISG15; blocks UBE1L E1 activation; sequesters ISGylated NP; blocks ISG15 secretion	(262–264)
	Vaccinia virus	E3	Binds and antagonizes human and mouse ISG15	(265)
	Rotavirus	NSP5	Targets UBE1L for proteasomal degradation	(266)
	HCMV	IE1	Downregulates ISG15 transcription	(267)
**MX proteins (MXA/MXB)** *Dynamin-like GTPases; trap viral nucleocapsids and polymerase subunits; MXB binds HIV-1 capsid and blocks nuclear import of viral cDNA; porcine MX1 blocks PRRSV N-Nsp9 interaction*	Influenza A virus	NP (mutant) †	Escape mutations in NP confer MXA resistance and host adaptation (1918 H1N1, H5N1, H7N7)	(269–273)
	HIV-1	Capsid (mutant) †	Capsid mutations (e.g., G208R and the CypA-binding loop) prevent MXB binding and escape restriction	(277–279)
	HIV-1	Rev (upregulated) †	Increased multiply-spliced RNA and Rev expression overcome MXB restriction	(280)
	HSV-1 (alphaherpesviruses)	Tegument packaging ‡	MxB is packaged into progeny virions and binds capsids via its N-terminal domain; role in restriction unclear	(83–85)
**OAS/RNase L** *OAS synthesizes 2’-5’-oligoadenylates (2-5A) that activate RNase L to cleave viral RNA at UA/UU dinucleotides; cleavage products amplify MDA5 signaling*	Influenza A virus	NS1	Sequesters dsRNA, preventing OAS activation	(281, 282)
	Vaccinia virus	E3L; D9/D10	dsRNA sequestration; decapping reduces dsRNA accumulation	(283–288)
	Theiler’s virus	L*	Binds the ankyrin domain of RNase L	(289)
	MHV	ns2	Phosphodiesterase degrading 2-5A	(290–293)
	Rotavirus	VP3	Phosphodiesterase degrading 2-5A	(291, 294)
	SARS-CoV-2	nsp15 (EndoU)	Cleaves viral RNA to limit dsRNA accumulation	(296, 297)
	HCV	Genome †	Interferon-resistant genotypes carry fewer UA/UU dinucleotides	(298)
	Poliovirus	ciRNA	RNA structure competitively inhibits RNase L	(299, 300)
	HIV	Tat	Binds TAR, preventing OAS activation by TAR	(301)
	HIV; EMCV	ABCE1 (host)	Virus-induced RNase L inhibitor	(302, 303)
**PKR** *dsRNA-triggered dimerization and trans-phosphorylation; phosphorylates eIF2α to halt translation; promotes stress granules and inflammasome activation*	Influenza A virus	NS1	Sequesters dsRNA; directly binds PKR via a five-residue peptide	(282, 305–307)
	Vaccinia virus	E3L; K3L	dsRNA sequestration and direct PKR binding; K3L acts as eIF2α pseudosubstrate	(308–310)
	HSV-1	ICP34.5	Recruits PP1 to dephosphorylate eIF2α	(311)
	HCV	NS5A (ISDR)	Directly binds PKR and disrupts dimerization	(312, 313)
	Adenovirus	VA-I RNA	dsRNA decoy; locks PKR in an inactive monomeric form	(314–316)
	EBV	EBER1	RNA decoy; weak PKR binding and activation	(317–319)
**IFIT family (ISG56/IFIT1, IFIT2, IFIT3, IFIT5)** *Bind non-self RNAs (cap0, 5’-ppp) via positively charged pockets and block translation initiation; IFIT2-IFIT3 heterodimers recognize viral mRNA 5’ ends*	Flaviviruses (e.g., WNV)	NS5 2’-O-methyltransferase	cap0→cap1 methylation protects viral RNA from IFIT1	(320–323)
	Coronaviruses (MHV, SARS-CoV, SARS-CoV-2)	nsp16/nsp10 2’-O-methyltransferase	cap-1 methylation evades IFIT1-mediated restriction	(324–327)
	Alphaviruses	5’ stem-loop (5’SL) †	RNA structure hides the cap0 from IFIT1 recognition	(328, 329)
	VEEV	5’ hairpin †	Hairpin structure prevents IFIT1 access to the cap	(330)
**Viperin** *Radical-SAM enzyme producing ddhCTP, a chain-terminating nucleotide inhibiting viral RdRPs; blocks viral budding via lipid rafts; enhances STING/TBK1 signaling*	HCV	NS5A	Binds viperin and blocks ddhCTP synthesis	(331)
	JEV	Host proteasome	Induces proteasome-mediated viperin degradation	(332)
	HCMV	Host relocalization ‡	Relocalizes viperin from ER to mitochondria; exploits it to reduce ATP generation	(333, 334)
	HIV-1	Proviral exploitation ‡	Viperin supports sustained infection in macrophages	(335, 336)
**PML/PML-NBs** *Nuclear bodies sequester viral proteins (e.g., EMCV 3Dpol); SUMO E3 ligase activity; LLPS-driven condensates; restrict DNA/RNA viruses and HIV-1 latency*	LCMV; Lassa virus	Z	Redistribute PML to the cytoplasm	(337)
	EV71	3Cpro	Proteasome-independent PML degradation	(193)
	EMCV	3Cpro	Cleaves PML	(191)
	Zika virus	NS5	SUMOylated NS5 disrupts PML-STAT2 NBs and directs PML degradation	(338)
	Dengue virus	NS5	Complexes with PML-III/IV to induce PML-NB breakdown	(339)
	HCMV	IE1	Binds PML coiled-coil; blocks PML SUMOylation (but see caveat, ref 342)	(340–342)
	HSV-1	ICP0	RING-type E3 ligase; proteasomal degradation of PML (isoform-selective)	(187, 343, 344)
	VZV	ORF61	ICP0 homolog disrupting PML-NBs	(345)
	Adenovirus	E4-ORF3	Polymer scaffold sequesters SUMO	(346–348)
	PRRSV	nsp1β	Inhibits PML expression at the post-translational level	(188)
	Influenza A virus	NS1	Binds TRIM25 and blocks its association with RIG-I, preventing TRIM25-mediated RIG-I ubiquitination	(349)
	PRRSV	nsp1α	Targets TRIM25 for proteasomal degradation and suppresses host antiviral responses	(350)
	PRRSV	miR-376b-3p (host)	Virus-upregulated miR-376b-3p targets TRIM22, countering TRIM22-mediated restriction of PRRSV	(351, 352)
**APOBEC3 family (APOBEC3A-H)** *Cytidine deaminases that deaminate single-stranded DNA; induce G-to-A hypermutation of reverse-transcribed viral DNA; restrict retroviruses, HBV and herpesviruses*	HIV-1	Vif	Recruits the CBFβ-CUL5-RBX2-ELOB-ELOC E3 ligase complex to degrade APOBEC3D, APOBEC3F, APOBEC3G, and APOBEC3H and block their incorporation into virions	(201–204, 353–357)
	Primate and feline lentiviruses	Vif; FIV protease	Species-specific degradation of host APOBEC3; FIV protease also antagonizes feline APOBEC3	(361, 362)
	Foamy virus	Bet	Binds APOBEC3 and impairs its dimerization and cytosolic solubility	(363, 364)
	Murine leukemia virus	Glycosylated Gag	Blocks APOBEC3 and cytosolic sensor access to the reverse transcription complex	(365, 366)
	EBV	BORF2 (RNR large subunit)	Inhibits APOBEC3B to preserve viral genome integrity	(367)
	HSV-1; HSV-2; EBV; KSHV	RNR large subunits	Conserved relocalization of APOBEC3B from the nucleus to the cytoplasm	(368–370)
	KSHV	ORF61	Sequesters APOBEC3B in filamentous cytoplasmic aggregates	(371)
	HCMV	RNR-independent mechanism	Mediates APOBEC3B relocalization early during infection	(372)
	HHV-6B	U28	Binds multiple APOBEC3 proteins and promotes their relocalization and degradation; HHV-6A lacks this activity and accumulates mutations	(373)
**IFITM family (IFITM1-3)** *Intramembrane proteins of endosomal and lysosomal membranes; block fusion of enveloped viruses; also used as entry factors by some coronaviruses*	HIV-1	Nef	Reduces IFITM3 incorporation into virions and restores fusion; depletes IFITM1–3 from extracellular vesicles	(374, 375)
	HIV-1 (transmitted variants)	Mutation †	Transmitted variants resist IFITM-mediated restriction; IFITM orthologs differ in anti-HIV-1 activity	(376, 377)
	HBV (host-mediated)	ABHD16A depalmitoylase ‡	Removes S-palmitoyl groups required for IFITM clustering, attenuating IFITM antiviral activity and the anti-HBV activity of IFITM1	(378, 379)
	HCoV-OC43; SARS-CoV-2; PEDV	Entry-factor exploitation ‡	IFITM2/IFITM3 used as entry factors; SARS-CoV-2 Spike hijacks IFITM1-3; YxxΦ motif mutations convert IFITM3 into an enhancer	(237–241)
**Tetherin (BST-2/CD317)** *GPI-anchored membrane protein that tethers budding virions at the plasma membrane and signals through NF-κB; restricts a broad range of enveloped viruses*	HIV-1	Vpu	Species-specific antagonism of human tetherin; depends on AP-1 and human-specific adaptations	(380–382)
	HIV-1 group M	Nef	Tetherin antagonism	(383)
	HIV-1 group O	Vpu variant	Tetherin antagonism	(384)
	HIV-2	Envelope glycoprotein	Incomplete BST-2 antagonism; modulates NF-κB signaling	(385)
	SIV	Envelope glycoprotein	Enhanced antagonism by a neurovirulent SIV	(386)
	FIV	Envelope glycoproteins; signal peptide	Distinctive mechanism requiring virion incorporation; the signal peptide itself antagonizes tetherin	(387, 388)
	EIAV	Envelope protein	Antagonizes equine tetherin	(389)
	Ebola virus	GP	Interacts with tetherin and can substitute for HIV-1 Vpu	(390)
	SARS-CoV; SARS-CoV-2	ORF7a	Blocks BST-2 glycosylation; neutralizes BST-2 at intracellular membranes; Omicron Spike enhances counteraction	(391–393)
	KSHV	K5	Ubiquitylates lysine residues in the tetherin N-terminal domain, leading to its degradation	(394)
	HSV-1	gM	Removes tetherin from the plasma membrane; can substitute for HIV-1 Vpu	(249)

†Escape mediated by viral mutation or RNA structure rather than by an encoded antagonist protein. ‡ Proviral exploitation of the ISG rather than classical antagonism. Reference numbers correspond to the manuscript reference list.

Meanwhile, studies on ISG function have fundamentally expanded our understanding of how viruses induce IFN production during infection. The traditional view holds that viral RNA species generated during replication trigger IFN induction non-discriminately, whereas certain viral proteins could block both IFN production and downstream signaling; the balance between these two opposing forces was thought to determine the IFN induction capacity of a given virus. However, recent advances demonstrate that even in the absence of alterations in classical viral IFN-antagonist proteins, the modification and recognition of viral RNA by both host ISGs and viral enzymes largely dictate the magnitude of IFN induction and viral replication fitness *in vivo*. The most compelling example is the IFIT-mediated discrimination of self-versus non-self RNA: the 2’-O-methyltransferase activity of flavivirus NS5 and coronavirus nsp16 converts viral cap-0 to cap-1, thereby shielding viral mRNA from IFIT1 recognition (321, 325, 327). West Nile virus lacking this methyltransferase activity is nonpathogenic in WT mice but reverts to lethality in *Ifit1*^−/−^ mice (321), demonstrating that a single viral enzyme targeting an ISG sensor, not a classical IFN-antagonist protein, determines *in vivo* virulence. Similarly, prokaryotic homologs of viperin discovered in bacteria and archaea produce antiviral nucleotide analogs (164), indicating that ISG-like antiviral strategies predate the IFN system and represent an ancient, conserved defense mechanism. These observations imply that viral proteins directly antagonizing ISG effector functions constitute a distinct and underexplored class of determinants of viral pathogenesis.

These insights have direct therapeutic implications. The discovery that ISGs regulate multiple nodes of the IFN response suggests that modulating ISG regulatory functions, rather than the IFN pathway itself, may offer more precise strategies for antiviral intervention. Furthermore, viral proteins that directly antagonize ISG effector functions, as systematically catalogued in this review, represent a class of antiviral drug targets complementary to those drugs directly targeting viral RNA replication or virion assembly, such as viral de-ISGylating proteases, dsRNA-binding proteins (influenza NS1, vaccinia E3L), and cap-methylating enzymes (coronavirus nsp16, flavivirus NS5). During the identification of tetherin’s antiviral function, an artificial tetherin-like protein was generated and shown to have anti-HIV activity (243). Recent advances in protein structure prediction and *de novo* design allow the generation of artificial proteins with designed functions rather than the use of proteins found in nature (413, 414). As the structures and mechanisms of the ISGs are investigated in more detail, artificial ISGs designed by *de novo* protein design tools could target conserved viral proteins or conserved steps in virus replication and be delivered as mRNA in lipid nanoparticles for transient *in vivo* expression (415). Such designed proteins would act directly on the virus rather than triggering a broad interferon response to avoid side effects during IFN treatment and the rise of mutants resistant to traditional antiviral drugs. We expect that further understanding of ISG function will yield novel strategies and mechanistic insights for host-directed antiviral therapeutics.
